# Variables Associated with Coronavirus Disease 2019 Vaccine Hesitancy Amongst Patients with Neurological Disorders

**DOI:** 10.3390/idr13030072

**Published:** 2021-08-30

**Authors:** Arash Ghaffari-Rafi, Kimberly Bergenholtz Teehera, Tate Justin Higashihara, Frances Tiffany Cava Morden, Connor Goo, Michelle Pang, Cori Xiu Yue Sutton, Kyung Moo Kim, Rachel Jane Lew, Kayti Luu, Shaina Yamashita, Catherine Mitchell, Enrique Carrazana, Jason Viereck, Kore Kai Liow

**Affiliations:** 1 Department of Neurological Surgery, School of Medicine, University of California Davis, Sacramento, CA 95817, USA; 2 John A. Burns School of Medicine, University of Hawai’i at Mānoa, Honolulu, HI 96813, USA; 3 University of Hawai’i at Mānoa, Honolulu, HI 96822, USA; 4 Brain Research, Innovation and Translation Lab, Hawaii Pacific Neuroscience, Honolulu, HI 96817, USA

**Keywords:** coronavirus disease 2019, neurological disorders, vaccine hesitancy, quality improvement, socioeconomic, demographic, risk factors, Hawaii

## Abstract

Introduction: Given that the success of vaccines against coronavirus disease 2019 (COVID-19) relies on herd immunity, identifying patients at risk for vaccine hesitancy is imperative—particularly for those at high risk for severe COVID-19 (i.e., minorities and patients with neurological disorders). Methods: Among patients from a large neuroscience institute in Hawaii, vaccine hesitancy was investigated in relation to over 30 sociodemographic variables and medical comorbidities, via a telephone quality improvement survey conducted between 23 January 2021 and 13 February 2021. Results: Vaccine willingness (*n* = 363) was 81.3%. Univariate analysis identified that the odds of vaccine acceptance reduced for patients who do not regard COVID-19 as a severe illness, are of younger age, have a lower Charlson Comorbidity Index, use illicit drugs, or carry Medicaid insurance. Multivariable logistic regression identified the best predictors of vaccine hesitancy to be: social media use to obtain COVID-19 information, concerns regarding vaccine safety, self-perception of a preexisting medical condition contraindicated with vaccination, not having received the annual influenza vaccine, having some high school education only, being a current smoker, and not having a prior cerebrovascular accident. Unique amongst males, a conservative political view strongly predicted vaccine hesitancy. Specifically for Asians, a higher body mass index, while for Native Hawaiians and other Pacific Islanders (NHPI), a positive depression screen, both reduced the odds of vaccine acceptance. Conclusion: Upon identifying the variables associated with vaccine hesitancy amongst patients with neurological disorders, our clinic is now able to efficiently provide ancillary COVID-19 education to sub-populations at risk for vaccine hesitancy. While our results may be limited to the sub-population of patients with neurological disorders, the findings nonetheless provide valuable insight to understanding vaccine hesitancy.

## 1. Introduction

While the United States (US) Federal Drug Administration (FDA) has approved several vaccines to address coronavirus disease 2019 (COVID-19), only an estimated 58–69% of US adults plan to get vaccinated [1]. Given that a vaccine’s success relies on extensive uptake within the community, there is impetus to conduct public outreach and vaccine education for patients at risk for vaccine hesitancy [2,3,4]. To efficiently address hesitancy, a comprehensive understanding of populations at risk across major sociodemographic and disease strata should first be developed.

Given Hawaii’s unique status as a minority-majority state, with the US’s largest share of multiracial citizens, the population serves as an ideal backdrop for identifying the drivers of vaccine hesitancy amongst historically underserved patients (i.e., Asians, Native Hawaiians and Other Pacific Islanders (NHPI), etc.) [5]. Moreover, regarding disease subsets, with neurological disorders being the leading cause of years of life lost and years lived with disability, as well as being associated with high risk for severe COVID-19, there should be heightened efforts to protect such a vulnerable subgroup [6,7,8]. Hence, to judiciously expend clinic resources in providing vaccine education and outreach, a quality improvement (QI) survey was conducted at a large Hawaii multidisciplinary neuroscience institution, with the goal of identifying the patient subsets at risk of vaccination hesitancy.

## 2. Methods

For this QI study, a telephone survey of Hawaii Pacific Neuroscience (HPN) adult (18 years and older) patients was conducted between 23 January 2021 and 13 February 2021 to identify populations at risk for COVID-19 vaccine hesitancy or declination—patient subsets requiring greater HPN clinic resources for vaccine counseling. Deemed a QI survey, institutional review board exemption was attained from the University of Hawai‘i at Mānoa, Office of Research Compliance. At survey onset, participants provided verbal informed consent after the disclosure of survey objectives, risks, and benefits, as well as assured anonymity; all data were deidentified. No incentive for participation or survey completion was provided. The survey followed reporting guidelines of the American Association for Public Opinion Research (https://www.aapor.org/Publications-Media/AAPOR-Journals/Standard-Definitions.aspx, accessed on 22 January 2021).

### 2.1. Survey Instrument

The survey was developed after consultation with a cross-functional work group of patients, clinicians, and ancillary healthcare providers. Survey questions emphasized sociodemographic and medical data readily attainable by HPN staff, from electronic medical records or via routine in-clinic pre-appointment questionnaires (i.e., surrogate variables which may readily identify high-risk patients for vaccine hesitancy/declination, therefore requiring time-investment by HPN for auxiliary COVID-19 vaccine counselling). The ten-minute survey explored variables potentially predictive of vaccine hesitancy, based on prior research or emerging speculation amongst the consulted work group [9,10,11,12]. 

Participants responded to a structured and scripted survey of 13 questions, including: whether the patient had been counselled on COVID-19 vaccination by a physician; the primary source of COIVD-19 information; perceptions of vaccine safety and severity of COVID-19 illness; whether the patient believes herself/himself to have a medical condition making COIVD-19 vaccination unsafe; history of annual influenza vaccination; history of testing positive for COVID-19; self-identified race/ethnicity; work status; highest level of education; marital status; and political views (Appendix A). Cases characterized as complete interviews required a 100% response rate to the crucial question (Do you plan on getting the COVID-19 vaccine?) and 80% for all other questions; partial interviews differed only in that 50–79% of other questions required responses; break-off was defined as either nonresponse to the crucial question or less than 50% response to all other questions [13]. Only data from complete and partial interviews were included for statistical analysis. Participants were provided with the opportunity to terminate the survey at any time and decline to answer any question. Primary caregivers were permitted to assist in participant interviews when appropriate. 

### 2.2. Study Population and Data Collection

Participants represented a random sample of the patients who had visited HPN at least once between 1 January 2019 and 1 January 2021. With four campuses (Honolulu, Kailua, Waikele, and Kona), the entire state of Hawaii serves as the patient catchment area for HPN (one of state’s largest multidisciplinary neurosciences clinical care and research centers, with over 20,000 patient visits annually) [14,15]. Utilizing a 5% margin of error and 95% confidence interval, an optimal sample size of 361 was calculated [16]. A total of 1494 randomly selected patients were called, with 363 providing survey responses. 

For all participants telephoned, sociodemographic data were collected from the most recent patient visit’s electronic medical records. Variables included age, insurance type, race, sex (female or male), and Zone Improvement Plan (ZIP) code of the patient’s residence. By linking ZIP codes to data attained from the US Census Bureau, 2019 American Community Survey 5-Year Estimates (http://www.census.gov, accessed on 22 January 2021), ZIP code served as a proxy measure for median household income, the population size of the patient’s municipality, and estimates of poverty in the patient’s municipality (i.e., percentage of all people, 18–64 years, and 65 years and over, whose income in the past 12-months was below the poverty level). The population size of the patient’s ZIP code was converted into a geographic classification established by the US Census Bureau: populations of 50,000 or more people were designated as urban; less than 50,000 to at least 2500 as suburban; and less than 2500 as rural. Median household income was coded into income quartiles, with quartile cut-offs tabulated for the baseline HPN population. Participant insurance type (Medicare, Medicaid, private, or military insurance) was classified according to criteria of the Agency of Healthcare Research and Quality (Rockville, MD, USA) for the Healthcare Cost and Utilization Project (www.hcup-us.ahrq.gov, accessed on 22 January 2021) [17,18]. Self-identified race was stratified as: White, Black, Asian, NHPI, and Native American or Alaskan Native (NAAN)).

For participants who provided complete or partial surveys, comorbidity data from the most recent visit were collected. Cardiovascular variables included body mass index (BMI; $\mathrm{kg}/m^{2}$), dyslipidemia, diabetes mellitus (type I or II), hypertension, coronary artery disease or prior myocardial infraction, peripheral vascular disease, congestive heart failure, history of atrial fibrillation or flutter, cerebrovascular accident (stroke or transient ischemic attack), and smoking status. Smoking status was classified as never (less than 100 cigarettes over lifetime), current, or former (current/former: 100 or more cigarettes over lifetime), per the US Centers for Disease Control and Prevention (CDC), National Health Interview Survey, Adult Tobacco Use (https://www.cdc.gov/nchs/surveys.htm, accessed on 22 January 2021).

The psychiatric variables collected included: history of any Diagnostic and Statistical Manual of Mental Disorders 5th Edition disorder, alcohol use disorder, and illicit substance use disorder (i.e., methamphetamine, cocaine, heroin, ecstasy, opioids, hallucinogens, and marijuana) [19]. Patients were also characterized as having a positive or negative screen for depressive disorder and alcohol abuse/dependance. Depression screening was conducted via the Patient Health Questionier-2 (PHQ-2), a two-question module validated to assess depression; a score of three or greater was deemed positive, with major depressive disorder likely [20]. Alcohol drinking habits were assessed by the Alcohol Use Disorders Identification Test-Consumption (AUDIT-C)—a validated version of the World Health Organization’s ten-question screen for harmful drinking patterns; scores of at least three for women and at least four for men were deemed positive for harmful drinking [21,22,23,24,25,26,27]. PHQ-2 and AUDIT-C scores were available for all patients, from the most recent clinic visit, as the institute’s standard protocol requires these questionnaires to be completed during patient intake [15].

Comorbidity data for general medical conditions were also collected, including: peptic ulcer disease, liver disease (patients with cirrhosis), connective tissue disease, chronic pulmonary disease, hemiplegia, dementia, moderate/severe renal disease (severe: on dialysis, post-kidney transplant, or with uremia; moderate: creatinine > 3 mg/dL), history of solid tumor (localized or metastasized), autoimmune disease, thyroid disease, and musculoskeletal disorder. A cumulative comorbidity status was calculated for each participant, via the Charlson Comorbidity Index (CCI), which, accounting for the type and number of comorbidities, provides a patient’s estimated survival at 10 years [28,29,30].

### 2.3. Statistical Analysis

Primary analysis utilized nonparametric testing, as assumptions of normality were not met by quantile–quantile plots and histograms. Continuous variables were assessed by the independent Wilcoxon rank sum test, while categorical variables by either the Pearson’s chi-squared test or the Fisher’s exact test of independence, with Haldane–Anscombe correction [31,32,33,34,35]. Nonparametric continuous variables were presented as the median and interquartile range (IQR, 25th percentile and 75th percentile). Categorical data were expressed as the odds ratio with the 95th percentile confidence interval; for a particular variable’s strata, each odds of the odds ratio represented the odds of accepting vaccination compared to declining it. Univariate and multivariable logistic regression, with Firth’s correction, was performed to identify variables independently associated with vaccine acceptance [36]. After regression diagnostics, variables for the multivariable analysis were chosen by stepwise selection using the Akaike Information Criterion (AIC), with the final model selected by the McFadden’s pseudo-$R^{2}$ and the lowest AIC [37,38,39,40]. All tests were two-tailed and used an alpha level of 0.05 for deeming statistical significance. Analyses were conducted through R Statistical Software (R Foundation for Statistical Computing, Vienna, Austria) [41].

## 3. Results

### 3.1. General Sample Characteristics

**Figure 1:**
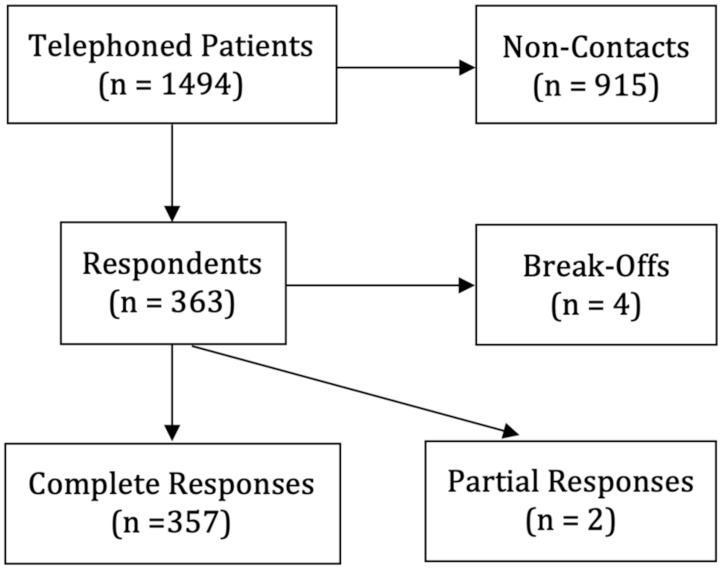
Sampled patients for survey.

From the 1494 randomly telephoned patients, 915 were non-contacts and 363 respondents (357 complete responses, two partial, and four break-offs; Figure 1). Including partial surveys, there was a response rate of 0.24, a cooperation rate of 0.62, a refusal rate of 0.147, and a contact rate of 0.388 [13]. Demographic breakdown of participants (complete and partial surveys) and non-participants can be found in Table S1.

### 3.2. Patients with Neurological Disorders: Entire Cohort

Between 23 January 2021 and 13 February 2021, 81.3% of HPN participants stated that they would accept a COVID-19 vaccination in the survey (Table 1). Patients accepting vaccination (61.50, IQR: 47.00, 72.00) were significantly older (7.00, 95% CI: 3.00, 12.00; *p* = 0.003). After stratification by sex and race, females declining vaccination were younger than male counterparts (Table 2, Table 3, Table 4, Table 5, Table 6 and Table 7). Patients on Medicaid had a significantly lower odds for vaccination (0.42, IQR: 0.22, 0.82; *p* = 0.007), while those from the third income quartile had greater odds for vaccination (2.31, IQR: 1.10, 5.33; *p* = 0.003).

#### 3.2.1. Medical Comorbidities

Participants with dyslipidemia (2.02, IQR: 1.11, 3.76; *p* = 0.021) or musculoskeletal disorder (1.92, IQR: 1.07, 3.49; *p* = 0.027) were at significantly increased odds for vaccination (Table 6). Meanwhile, drug use was associated with a significantly decreased odds for vaccine acceptance (0.32; IQR: 0.11, 0.96; *p* = 0.030). Overall, patients with higher Charlson Comorbidity Index (CCI) scores (i.e., lower 10-year survival estimates [%]) were more likely to accept vaccination (*p* = 0.002).

#### 3.2.2. Survey Responses

Participants whose primary source of COVID-19 information was from traditional media had a greater odds of vaccine acceptance (1.82, IQR: 1.02, 3.28; *p* = 0.042), contrary to those whose primary source was social media (0.26, IQR: 0.11, 0.63; *p* = 0.001; Table 2 and Table 3). Odds of vaccine acceptance were significantly lower for those perceiving the vaccine as not safe (0.087, IQR: 0.038, 0.19; *p* < 0.001) or COVID-19 as not a severe illness (0.21, IQR: 0.094, 0.49; *p* < 0.001). Patients with a self-perception of a preexisting medical condition believed to make the vaccine unsafe were also at reduced odds for vaccine acceptance (0.20, IQR: 0.11, 0.37; *p* < 0.001). Those who did not receive the influenza vaccine within the past year had reduced odds of COVID-19 vaccine acceptance (0.20, IQR: 0.11, 0.36; *p* < 0.001). If not able to work, the odds of vaccine acceptance were significantly lower (0.46, IQR: 0.23, 0.93; *p* = 0.026). Participants with only a high school degree had lower odds of vaccine acceptance (0.37, IQR: 0.20, 0.71; *p* = 0.002), while those with a graduate degree had increased odds (3.60, IQR: 1.25, 14.19; *p* = 0.01). Regarding political views, political liberals had increased odds of vaccine acceptance (2.20, IQR: 1.02, 5.18; *p* = 0.048). Relative to Whites, Hispanics (0.30, 95% CI: 0.090, 0.97; *p* = 0.044) and NHPIs (0.48, 95% CI: 0.24, 0.95; *p* = 0.034) had significantly decreased odds for vaccine acceptance (Table 8 and Table 9).

#### 3.2.3. Multivariable Logistic Regression

Multivariable analysis identified additional predictors of vaccination (Table 8 and Table 9). Patients who were current smokers had lower odds of vaccination (0.22, 95% CI: 0.049, 0.95; *p* = 0.042). Meanwhile, patients who had undergone a cerebrovascular accident had increased odds for vaccine acceptance (24.75, 95% CI: 1.84, 333.64; *p* = 0.016).

### 3.3. Female Patients

When examining female participants (Table 2, Table 4 and Table 6), regarding political views, those identifying as liberal presented with the greatest odds for vaccine acceptance (4.10, 95% CI: 1.30, 17.18; *p* = 0.009).

### 3.4. Male Patients

When examining male participants (Table 2, Table 4 and Table 6), insurance, race, history of a solid tumor, BMI, and illicit drug use were uniquely significant. Males identifying as NHPIs had decreased odds (0.34, 95% CI: 0.13, 0.89; *p* = 0.022) for vaccine acceptance. Meanwhile, males with a history of a solid tumor had a decreased odds (0.22, 95% CI: 0.057, 0.87; *p* = 0.019). From the multivariable analysis (Table 8 and Table 9), political conservatives had significantly decreased odds for vaccine acceptance (0.00, 95% CI: 0.00, 0.50; *p* = 0.034).

### 3.5. White Patients

Amongst white patients (Table 3, Table 5 and Table 7), those with illicit drug use (0.19, 95% CI: 0.049, 0.75; *p* = 0.009) presented with significantly decreased odds of vaccine acceptance.

### 3.6. Asian Patients

Amongst Asian patients (Table 3, Table 5 and Table 7), insurance and BMI were identified as statistically significant variables. Asians with military insurance were at decreased odds of vaccine acceptance (0.055, 95% CI: 0.0011, 0.57; *p* = 0.005). Meanwhile, in multivariable analysis, the adjusted odds of BMI (0.88, 95% CI: 0.78, 0.99; *p* = 0.032) was lower for patients accepting vaccination.

### 3.7. Native Hawaii or Other Pacific Islander Patients

For NHPI patients (Table 3, Table 5, Table 7 and Table 9), the PHQ-2 depression screen and history of a solid tumor were identified as statistically significant variables. NHPIs with positive depression screen (0.12, 95% CI: 0.016, 0.76; *p* = 0.010) or solid tumor (0.15, 95% CI: 0.020, 0.89; *p* = 0.017) were at decreased odds of vaccine acceptance.

## 4. Discussion

### 4.1. Patients with Neurological Disorders: Entire Cohort

To judiciously allocate clinic resources for COVID-19 vaccine counseling, our neuroscience center sought to first identify patient populations exhibiting vaccine hesitancy. From the 359 patients with neurological disorders surveyed, 81.3% accepted vaccination in our cohort. Fifteen variables were found to be associated with vaccine hesitancy: age, insurance type, income quartile, dyslipidemia, illicit drug use, the presence of a musculoskeletal disorder, CCI, employment status, education level, political views, annual influenza vaccination status, source of COVID-19 information, perception of COVID-19’s illness severity, concerns about vaccine safety, and apprehension regarding a preexisting medical condition adversely interacting with the vaccine.

#### 4.1.1. Race

Although the general cohort analyses did not reveal trends regarding race, subgroup analysis did. Male NHPI patients were at reduced odds of vaccination, while themselves, NHPI patients with a positive depression screen or history of a solid tumor were at reduced odds for vaccination. Given the inherent health disparities secondary to structural inequalities, enhanced outreach efforts should be extended to NHPI patients to ensure equitable opportunities for vaccination, particularly amongst those who are PHQ-2 positive, with a tumor history, or male [42,43].

#### 4.1.2. Age

While patients with vaccine hesitancy were overall significantly younger, upon stratification, the trend was only observed amongst females and Whites. Hesitancy amongst younger females may reflect concerns regarding the COVID-19 vaccine adversely interacting with pregnancy, given the population’s lack of inclusion in COVID-19 vaccine clinical trials—in spite of recommendations that the vaccination is not withheld from pregnant patients [44,45,46,47,48,49]. Therefore, amongst young patients with neurological disorders, those who are female or White should be targeted for vaccine counseling if appropriate.

#### 4.1.3. Insurance Type

Likewise, Medicaid patients exhibited reduced odds of COVID-19 vaccination, paralleling observed trends for other vaccines, where patients on public insurance have reduced vaccination rates [50,51]. Medicaid patients represent a financially disadvantaged population, who experience reduced healthcare utilization secondary to not affording copayments, hence the lower COVID-19 vaccination odds may arise from financial concerns [52,53]. Upon demographic stratification, only amongst male Medicaid beneficiaries was vaccine acceptance reduced. Consequently, emphasizing the absence of cost for COVID-19 vaccination amongst the Medicaid population—with particular focus on males—could increase vaccine acceptance amongst the community.

Further subgroup analyses demonstrated trends amongst females and Asian patients. For females, Medicare patients were found to have significantly greater odds of vaccination, while among Asian patients, those with military insurance had reduced odds of vaccination, corresponding with reports of greater vaccine hesitancy amongst military personnel, arising secondary to distrust of the vaccine development process and concerns regarding vaccine safety [54]. Therefore, to increase vaccine acceptance amongst military members of Asian heritage, more resources should be expended to educate about the safety of the COVID-19 vaccine and authenticity of the FDA approval process.

#### 4.1.4. Income Quartile, Work Status, and Education Level

In contrast, patients in the third income quartile—the historical middle class—exhibited the greatest odds of vaccination [55]. The third quartile likely represents patients not only with greater COVID-19 exposure risk (i.e., work in healthcare or in contact with the general public), but also greater health literacy and reduced barriers to vaccination [56,57]. Accordingly, neurological patients not able to work (males particularly), thus having greater likelihood of isolation from the general public, or with only a high school education (specifically Whites and both sexes), were at reduced odds of vaccination, while those with a graduate degree exhibited greater odds of vaccine acceptance (particularly females and Whites). Therefore, limited resources would likely be best expended on counseling patients not able to work or with only a high school education.

#### 4.1.5. Information Source: Traditional Media and Social Media

As education level and health literacy correlate with ability to discern misinformation, patients acquiring knowledge from sources prone to false information may be less inclined to vaccinate [57,58,59]. Indeed, patients—males in particular—utilizing social media as a primary source of COVID-19 information had reduced odds of vaccination, contrary to those relying on traditional media. Given the pervasiveness of misinformation on social media and that social media use is highly predictive for believing vaccines are unsafe, clinicians should seek to address a patient’s false misconceptions or direct patients towards reputable information sources [57,58,59,60].

#### 4.1.6. Concerns of Vaccine Safety and Adverse Interaction with Preexisting Medical Conditions

Notwithstanding the information source, concerns regarding vaccine safety independently yielded a significantly reduced odds of vaccine acceptance; vaccine safety was the only variable to be statistically significant amongst all demographic strata (females, males, NHPIs, Asians, and Whites). Likewise, patients with the self-perception of a medical condition making vaccination unsafe (most evident amongst both sexes, Asians, and Whites) were at significantly reduced odds of vaccine acceptance, despite the CDC Advisory Committee on Immunization Practices (ACIP) authorizing COVID-19 vaccination for those with underlying medical conditions without contraindications (i.e., immediate allergic reaction to any vaccine components or severe allergic reaction to first dose) [61]. Hence, public health campaigns and physician counseling sessions should focus on alleviating vaccine safety concerns, as well as any individual patient concerns on vaccine interaction with suspected preexisting medical conditions.

#### 4.1.7. Medical Comorbidities

While there were reduced odds of vaccine acceptance amongst those with the self-perception of a preexisting medical condition making vaccines unsafe, patients with more clinically diagnosed comorbidities (per CCI) were at greater odds for vaccine acceptance—in particular, amongst females and Whites. Independently, patients (Whites specifically) with dyslipidemia or musculoskeletal disorders were at greater odds for vaccination; while among Whites alone, hypertension increased odds of vaccine acceptance. Patients with dyslipidemia and hypertension potentially represent a cohort of patients who are already engaged in preventative practices (i.e., diet modification, statins, anti-hypertensives, etc.), and thus have an appreciation for the benefits that preventative healthcare can provide; these patients are therefore more willing to vaccinate against a preventable illness. Similarly, patients with musculoskeletal disorders suffer from physically debilitating illnesses which often impactfully respond to medications or lifestyle modifications, and therefore greater vaccination acceptance may represent these patients’ first-hand positive experiences with healthcare interventions.

Overall, healthier patients (lower CCI) are potentially demonstrating vaccine complacency, where the risk of vaccine-preventable disease is perceived as low and vaccines are therefore viewed as unnecessary [2]. Within the HPN cohort, the role of vaccine complacency was directly demonstrated, in that patients who did not believe COVID-19 to be a severe illness exhibited reduced odds of vaccination—in subgroup analyses, such was most evident amongst Whites, as well as both sexes. Therefore, vaccine education efforts should address vaccine complacency, particularly amongst healthier patients.

In contrast to the trend of patients with greater illness severity seeking vaccination, amongst the male subgroup, history of a solid tumor reduced odds of vaccine acceptance (likewise observed amongst NHPI patients). In studies of hematological cancers, vaccine hesitancy was most attributed to concerns that the vaccines were not appropriately tested among cancer patients, notwithstanding expert oncologist opinions advocating vaccination and the CDC omitting cancer as a contraindication [61,62]. Therefore, greater outreach should be undertaken by oncologists to advocate vaccination if appropriate, by allaying misconceptions amongst cancer patients [62].

#### 4.1.8. Illicit Drug Use

Contrary to the other medical comorbidities, patients with illicit drug use (Whites specifically) demonstrated reduced odds of vaccine acceptance. Similar trends have been observed for the influenza vaccine and cancer screening, where patients with substance abuse disorder are less likely to attain preventative healthcare [63,64,65,66]. As a marginalized population, patients with illicit drug use are often detached from and mistrust the healthcare system—by extension, these patients may be more reliant on illegitimate information sources [67,68]. Therefore, when counseling patients with illicit drug use, emphasis should be placed on building trust and providing accurate COVID-19 information [67].

#### 4.1.9. Influenza Vaccination Status

One variable which can be efficiently extracted from electronic medical records to identify patients requiring COVID-19 vaccine counseling is annual influenza vaccine status. Patients who did not receive the influenza vaccine in the past year were at reduced odds for COVID-19 vaccine acceptance (the trend evident amongst both sexes, Asians, and Whites). Hence, predictors of influenza vaccine hesitancy may be similar to those of COVID-19, including vaccine complacency or concerns regarding side effects [2,69].

#### 4.1.10. Political Views

Regarding political views, neurological patients identifying as liberal were at greater odds for vaccine acceptance—amongst subgroup analyses, the trend was notable only among females. Such results demonstrate that, even in Hawaii, one of the more liberal states, COVID-19 vaccine acceptance remains highly politicized—as with the rest of the nation [70]. Therefore, patient education should seek to utilize neural apolitical sources for vaccine endorsement [70].

### 4.2. Strongest Predictors of Vaccine Acceptance

After conducting the univariate analysis, multivariable logistic regression was utilized to identify the strongest predictors of vaccine acceptance. For the overall cohort, seven variables were recognized: primary information source (social media use), concerns regarding vaccine safety, belief of COVID-19 to be a severe illness, self-perception of having a pre-existing medical condition making vaccination unsafe, education level (some high school), smoking status (current smoker), and history of a cerebrovascular accident. Current smokers and patients without a high school diploma were identified as having reduced odds of vaccination, and thus such populations require targeted intervention to mitigate potential health disparities from a lack of vaccination [71]. In contrast, patients who had experienced a cerebrovascular accident had greater odds of vaccine acceptance—these patients may be more inclined to engage with preventable health measures, secondary to having personally suffered a potentially avoidable life-altering illness [72].

Upon subgroup analysis, several unique trends were identified. After multivariable regression, only amongst females, Whites, and Asians, did concerns relating to vaccine safety, as well as self-perception of having a preexisting medical condition making vaccination unsafe, result in reduced odds of vaccine acceptance—therefore, if focused demographic-oriented interventions are applied, education on vaccine safety and side effects may be most impactful for females, Whites, and Asians. For both sexes, not having received the annual influenza vaccine significantly reduced odds of COVID-19 vaccine acceptance, yet only amongst males did the perception of COVID-19 as a non-severe illness or those identifying as politically conservative significantly reduce odds. Meanwhile, there were several variables resulting in reduced odds of vaccine acceptance, which were exclusive to certain racial groups: for Whites, having only a high school degree; for Asians, a greater BMI; and for NHPIs, a positive depression screen. Therefore, to conduct public outreach or patient counseling efficiently, there may be utility in focusing on specific variables depending on the sociodemographic group of interest.

### 4.3. Limitations

While the findings of this investigation may be extrapolated to other subpopulations, the results should be considered in the context of several limitations, including subset sample sizes. Inherently, generalizability could be restricted, as our population represents patients with neurological disorders from a single institution, as well as from a minority-majority state with unique sociocultural dynamics and differentially impacted by COVID-19. In Hawaii specifically, around the time of the survey (1 February 2021), the state had reported a cumulative 25,943 cases, 410 deaths, and 5.1% of the population had been vaccinated [73]. On 1 February 2021, there were 91 new cases, zero deaths within seven days, and a 2.1% positivity rate [73]. Given the lower rates of new cases at the time of surveying (relative to 21 August 2021: 671 daily cases, 8.3% test positivity, and nine new deaths within seven days) and the progression of COVID-19, our results may underestimate the current percentage of patients seeking vaccination [73,74]. Likewise, the variables representing disease hesitancy at the time of surveying may also have shifted and be dependent on daily case numbers and deaths [73,74].

Moreover, as participation was restricted to patients with a phone, the results may have been influenced by selection bias, in that more vulnerable subgroups (i.e., financially disadvantaged) could have been excluded. Finally, given the potentially polarizing nature of some survey questions, social desirability bias may have yielded participants providing responses which would be extrinsically viewed positively by others.

## 5. Conclusions

This QI survey provided our institution with actionable data, permitting for the efficient utilization of limited clinic resources, in providing vaccine counseling to at-risk patients with neurological disorders. In particular, patients with the following characteristics were recognized for being at risk of vaccine hesitancy (Table 10 and Table 11): not having received an annual influenza vaccine, a younger age, a higher CCI, illicit drug use, Medicaid insurance, and social media use for COVID-19 information. Meanwhile, uniquely reducing odds were observed amongst Whites, Asians, and NHPIs, for the following respective variables: high school degree, military insurance, and a positive depression screen. Moreover, amongst all subgroups, vaccine hesitancy appears to be associated with concerns that vaccines are not safe and the self-perception of a preexisting medical condition making the vaccine unsafe (expect among NHPIs). Therefore, focused counseling on allaying patient fears of comorbidity contraindications or vaccine safety may be most impactful. In summary, the investigation not only identified variables that increase the odds of vaccine hesitancy, but also recognized that amongst different demographic strata, there are unique variables at play.

## Figures and Tables

**Table 1:** Number of patients stratified by sociodemographic variables and comorbidities.

	Total Participants per Category (Acceptance of Vaccine/Total Participants in Strata)					
	All Patients	Female	Male	White	Asian	NHPI
Age	292/359 (81.3%)	158/195 (81.0%)	134/164 (81.7%)	128/149 (85.9%)	79/97 (81.4%)	58/78 (74.4%)
Sex						
Female	158/195 (81.0%)			73/86 (84.9%)	40/53 (75.5%)	30/38 (78.9%)
Male	134/164 (81.7%)			55/63 (87.3%)	39/44 (88.6%)	28/40 (70.0%)
Median Household Income	287/353 (81.3%)	155/191 (81.2%)	132/162 (81.4%)	126/147 (85.7%)	78/96 (81.3%)	57/77 (74.0%)
Overall Poverty Level in Municipality	287/353 (81.3%)	155/191 (81.2%)	132/162 (81.4%)	126/147 (85.7%)	78/96 (81.3%)	57/77 (74.0%)
Poverty Level for Ages 18–64	287/353 (81.3%)	155/191 (81.2%)	132/162 (81.4%)	126/147 (85.7%)	78/96 (81.3%)	57/77 (74.0%)
Poverty Level for Ages 65 and Older	287/353 (81.3%)	155/191 (81.2%)	132/162 (81.4%)	126/147 (85.7%)	78/96 (81.3%)	57/77 (74.0%)
Geographic Origin Population Size	287/353 (81.3%)	155/191 (81.2%)	132/162 (81.4%)	126/147 (85.7%)	78/96 (81.3%)	57/77 (74.0%)
Geographic Origin						
Urban	153/190 (80.5%)	87/107 (81.3%)	66/83 (79.5%)	66/76 (86.8%)	47/59 (79.7%)	28/40 (70.0%)
Suburban	129/156 (82.7%)	64/79 (81.0%)	65/77 (84.4%)	57/67 (85.1%)	31/37 (83.8%)	28/36 (77.8%)
Rural	5/7 (71.4%)	4/5 (80.0%)	1/2 (50.0%)	3/4 (75.0%)	0/0 (NA)	1/1 (100%)
Insurance Type						
Medicare	87/101 (86.1%)	42/46 (91.3%)	45/55 (81.8%)	44/49 (89.8%)	26/30 (86.7%)	13/17 (76.5%)
Medicaid	46/67 (68.7%)	24/34 (70.6%)	22/33 (66.7%)	15/21 (71.4%)	14/17 (82.4%)	16/25 (64.0%)
Private	114/140 (81.4%)	70/88 (79.5%)	44/52 (84.6%)	44/53 (83.0%)	35/44 (79.5%)	25/31 (80.6%)
Military	38/44 (86.4%)	18/23 (78.3%)	20/21 (95.2%)	24/25 (96.0%)	0/2 (0.0%)	3/4 (75.0%)
Income Quartiles						
Quartile 1	71/88 (80.7%)	33/41 (80.5%)	38/47 (80.9%)	31/34 (91.2%)	19/23 (82.6%)	13/19 (68.4%)
Quartile 2	70/88 (79.5%)	38/48 (79.2%)	32/40 (80.0%)	24/30 (80.0%)	18/24 (75.0%)	22/28 (78.6%)
Quartile 3	84/94 (89.4%)	49/55 (89.0%)	35/39 (89.7%)	45/49 (91.8%)	22/24 (91.7%)	9/1 (81.9%)
Quartile 4	62/83 (74.7%)	35/47 (74.5%)	27/36 (75.0%)	26/34 (76.5%)	19/25 (76.0%)	13/19 (68.4%)
**Survey Questions**						
	**All Patients**	**Female**	**Male**	**White**	**Asian**	**NHPI**
Q1: Have you had a one-on-one discussion with a physician about the risks and benefits of receiving the COVID vaccination?						
Had Conversation	67/79 (84.8%)	44/49 (89.8%)	23/30 (76.7%)	30/33 (90.9%)	18/21 (85.7%)	11/13 (84.7%)
No Conversation	225/280 (80.4%)	114/146 (78.1%)	111/134 (82.8%)	98/116 (84.5%)	61/76 (80.3%)	47/65 (72.3%)
Q2: What is your primary source of COVID information?						
Scholarly Articles/CDC/ US Governmental Agencies	56/66 (84.8%)	35/42 (83.3%)	21/24 (87.5%)	31/34 (91.2%)	4/6 (66.7%)	10/12 (83.3%)
Friends/Family/Coworkers	34/46 (73.9%)	21/26 (80.8%)	13/20 (65.0%)	13/16 (81.3%)	9/11 (81.8%)	9/15 (60.0%)
Healthcare Provider	13/15 (86.7%)	7/9 (77.8%)	6/6 (100%)	6/7 (85.7%)	3/4 (75.0%)	4/4 (100%)
Traditional Media	169/197 (85.8%)	82/98 (83.7%)	87/99 (87.9%)	71/81 (87.7%)	53/61 (86.9%)	33/41 (80.5%)
Social Media	16/28 (57.1%)	10/16 (62.5%)	6/12 (50.0%)	5/8 (62.5%)	9/13 (69.2%)	2/5 (40.0%)
Q3: Do you believe that vaccines are safe?						
Safe	270/310 (87.1%)	146/169 (86.4%)	124/141 (87.9%)	119/132 (90.2%)	75/87 (86.2%)	51/61 (83.6%)
Not Safe	14/38 (36.8%)	7/20 (35.0%)	7/18 (38.9%)	6/13 (46.2%)	2/7 (28.6%)	5/14 (35.7%)
Q4: Do you believe that COVID is a severe illness?						
Severe	271/322 (84.2%)]	146/174 (83.9%)	125/148 (84.5%)	123/138 (89.1%)	71/87 (81.6%)	52/66 (78.8%)
Not Severe	17/32 (53.1%)]	10/19 (52.6%)	7/13 (53.8%)	4/10 (40.0%)	7/8 (87.5%)	5/11 (45.5%)
Q5: Do you have a preexisting medical condition that you believe will make the vaccine unsafe?						
Preexisting Condition	44/75 (58.7%)	25/44 (56.8%)	19/31 (61.3%)	16/27 (59.3%)	10/20 (50.0%)	15/23 (65.2%)
No Preexisting Condition	237/270 (87.8%)	126/142 (88.7%)	111/128 (86.7%)	110/118 (93.2%)	65/73 (89.0%)	40/51 (78.4%)
Q6: Have you received the flu vaccine within the last year?						
Received Flu Shot	212/235 (90.2%)	121/134 (90.3%)	91/101 (90.1%)	91/96 (94.8%)	61/68 (89.7%)	41/51 (80.4%)
Did Not Receive Flu Shot	78/121 (64.5%)	35/58 (60.3%)	43/63 (68.3%)	36/52 (69.2%)	17/27 (63.0%)	17/27 (63.0%)
Q7: Have you tested positive for COVID?						
Tested Positive	4/6 (66.7%)	3/4 (75.0%)	1/2 (50.0%)	0/1 (0.0%)	1/2 (0.0%)	2/2 (100%)
Denied Positive Test	287/351 (81.8%)	154/190 (81.1%)	133/161 (82.6%)	128/148 (86.5%)	77/94 (81.9%)	56/76 (73.7%)
Q8: With a single category, how would you define your race/ethnicity?						
White	128/149 (85.9%)	73/86 (84.9%)	55/63 (87.3%)			
Black	9/9 (100%)	2/2 (100%)	7/7 (100%)			
Asian	79/97 (81.4%)	40/53 (75.5%)	39/44 (88.6%)			
Native Hawaiian/Other Pacific Islander	58/78 (74.4%)	30/38 (78.9%)	28/40 (70%)			
Hispanic	9/14 (64.3%)	6/9 (66.7%)	3/5 (60.0%)			
Native American or Alaskan Native	4/4 (100%)	4/4 (100%)	0/0 (NA)			
Q9: How would you define your work status?						
Employed	99/118 (83.9%)	52/65 (80.0%)	47/53 (88.7%)	40/46 (87.0%)	28/34 (82.4%)	20/24 (83.3%)
Homemaker	12/16 (75.0%)	11/15 (73.3%)	1/1 (100%)	10/12 (83.3%)	1/2 (50.0%)	1/1 (100)
Not Able to Work	39/56 (69.6%)	22/30 (73.3%)	17/26 (65.4%)	16/21 (76.2%)	4/7 (57.1%)	16/24 (67.7%)
Retired	116/138 (84.1%)	59/68 (86.8%)	57/70 (81.4%)	54/61 (88.5%)	38/45 (84.4%)	15/21 (71.4%)
Student	8/10 (80.0%)	5/7 (71.4%)	3/3 (100%)	1/1 (100%)	3/4 (75.0%)	2/3 (66.7%)
Unemployed	16/19 (84.2%)	8/9 (88.9%)	8/10 (80%)	7/8 (87.5%)	3/3 (100%)	4/5 (80.0%)
Q10: What is the highest level of education you completed?						
Graduate Degree	56/60 (93.3%)	26/27 (96.3%)	30/33 (90.9%)	35/36 (97.2%)	11/13 (84.6%)	2/2 (100%)
High School Degree	50/73 (68.5%)	23/34 (67.6%)	27/39 (69.2%)	11/19 (57.9%)	16/21 (76.2%)	21/28 (75.0%)
Some College	70/87 (80.5%)	38/49 (77.6%)	32/38 (84.2%)	29/34 (85.3%)	20/22 (90.9%)	11/19 (57.8%)
Some High School	12/15 (80.0%)	8/8 (100%)	4/7 (57.1%)	3/4 (75.0%)	4/4 (100%)	4/5 (80.0%)
Trade School	8/10 (80.0%)	5/6 (83.3%)	3/4 (75.0%)	5/5 (100%)	2/3 (66.7%)	1/2 (50.0%)
Associate/Bachelor’s Degree	93/108 (86.1%)	57/68 (83.8%)	36/40 (90.0%)	45/50 (90.0%)	25/32 (78.1%)	18/20 (90.0%)
Q11: What is your marital status?						
Divorced	39/49 (79.6%)	16/22 (72.7%)	23/27 (85.1%)	19/23 (82.6%)	6/9 (66.7%)	10/13 (76.9%)
Married	157/190 (82.6%)	81/99 (81.8%)	76/91 (83.5%)	74/83 (89.2%)	43/53 (81.1%)	24/33 (72.7%)
Single	66/85 (77.6%)	39/49 (79.6%)	27/36 (75.0%)	25/32 (78.1%)	18/21 (85.7%)	19/26 (73.1%)
Widowed	28/32 (87.5%)	22/25 (88.0%)	6/7 (85.7%)	9/10 (90.0%)	12/14 (85.7%)	5/6 (83.3%)
Q12: How would you describe your political view?						
Conservative	62/79 (78.5%)	28/38 (73.7%)	34/41 (82.9%)	30/36 (83.3%)	22/28 (78.6%)	5/9 (55.6%)
Independent	101/124 (81.5%)	49/63 (77.8%)	52/61 (85.2%)	45/53 (84.9%)	24/28 (85.7%)	24/33 (72.8%)
Liberal	90/100 (90.0%)	53/57 (93.0%)	37/43 (86.0%)	44/47 (93.6%)	20/21 (95.2%)	19/22 (86.4%)
**Comorbidities/Medical Conditions**						
	**All Patients**	**Female**	**Male**	**White**	**Asian**	**NHPI**
Body Mass Index	263/325 (80.9%)	140/178 (78.7%)	122/148 (82.4%)	117/138 (84.8%)	72/90 (80.0%)	51/67 (76.1%)
Dyslipidemia						
Dyslipidemia	140/162 (86.4%)	62/71 (87.3%)	78/91 (85.7%)	56/60 (93.3%)	42/51 (82.4%)	29/35 (82.9%)
No Dyslipidemia	129/170 (75.9%)	82/108 (75.9%)	47/62 (75.8%)	63/80 (78.8%)	30/39 (76.9%)	24/35 (68.6%)
Type 1 or 2 Diabetes Mellitus						
Diabetes Mellitus	44/53 (83.0%)	22/25 (88.0%)	22/28 (78.6%)	13/17 (76.5%)	17/18 (94.4%)	13/16 (81.3%)
No Diabetes Mellitus	225/279 (80.6%)	122/154 (79.2%)	103/125 (82.4%)	106/123 (86.2%)	55/72 (76.4%)	40/54 (74.1%)
Hypertension						
Hypertension	129/152 (84.9%)	63/73 (86.3%)	66/79 (83.5%)	47/50 (94.0%)	40/49 (81.6%)	31/40 (77.5%)
No Hypertension	140/180 (77.8%)	81/106 (76.4%)	59/74 (79.7%)	72/90 (80.0%)	32/41 (78.0%)	22/30 (73.3%)
Coronary Artery Disease or Prior Myocardial Infarction (CAD/MI)						
CAD/MI	25/33 (75.8%)	11/14 (78.6%)	14/19 (73.7%)	6/8 (75.0%)	11/13 (84.6%)	7/11 (63.6%)
No CAD/MI	244/299 (81.6%)	133/165 (80.6%)	111/134 (82.8%)	113/132 (85.6%)	61/77 (79.2%)	46/59 (78.0%)
Peripheral Vascular Disease (PVD)						
PVD	10/13 (77.0%)	6/6 (100%)	4/7 (57.1%)	2/2 (100%)	2/3 (66.7%)	6/8 (75.0%)
No PVD	259/319 (81.2%)	138/173 (79.8%)	121/146 (82.8%)	117/138 (84.8%)	70/87 (80.5%)	47/62 (75.8%)
Smoking Status						
Current Smoker	23/33 (70.0%)	13/19 (68.4%)	10/14 (71.4%)	9/14 (64.3%)	4/5 (80.0%)	8/12 (66.7%)
Former Smoker	46/55 (83.6%)	20/21 (95.2%)	26/34 (76.5%)	19/23 (82.6%)	12/15 (80.0%)	11/13 (84.6%)
Never Smoker	197/241 (81.7%)	110/138 (79.7%)	87/103 (84.5%)	90/102 (88.2%)	55/69 (79.7%)	34/45 (75.6%)
Congestive Heart Failure (CHF)						
CHF	6/7 (85.7%)	3/3 (100%)	3/4 (75.0%)	2/2 (100%)	1/2 (50.0%)	3/3 (100%)
No CHF	262/324 (80.9%)	141/176 (80.1%)	121/148 (81.8%)	117/138 (84.8%)	70/87 (80.5%)	50/67 (74.6%)
Atrial Fibrillation (Afib)						
Afib	21/24 (87.5%)	9/10 (90.0%)	12/14 (85.7%)	9/9 (100%)	4/5 (80.0%)	7/9 (77.8%)
No Afib	248/308 (80.5%)	135/169 (79.9%)	113/139 (81.3%)	110/131 (84.0%)	68/85 (80.0%)	46/61 (75.4%)
Cerebrovascular Accident (CVA)						
CVA	45/50 (90.0%)	23/24 (95.8%)	22/26 (84.6%)	19/19 (100%)	10/13 (76.9%)	14/16 (87.5%)
No CVA	224/282 (79.4%)	121/155 (78.1%)	103/127 (81.1%)	100/121 (82.6%)	62/77 (80.5%)	39/54 (72.2%)
Alcohol Use Screen						
Positive Screen	33/41 (80.5%)	15/21 (71.4%)	18/20 (90.0%)	19/24 (79.2%)	6/8 (75.0%)	4/4 (100%)
Negative Screen	234/289 (81.0%)	129/158 (81.6%)	105/131 (80.2%)	97/113 (85.8%)	66/82 (80.5%)	49/66 (74.2%)
Alcohol Use Disorder						
Alcohol Use Disorder	7/8 (87.5%)	0/1 (0.0%)	7/7 (100%)	5/6 (83.3%)	0/0 (NA)	2/2 (100%)
No Alcohol Use Disorder	257/319 (80.7%)	142/176 (80.7%)	115/143 (80.4%)	111/131 (84.7%)	71/89 (79.8%)	51/68 (75.0%)
Depression Screen						
Positive Screen	25/33 (75.8%)	12/15 (80.0%)	13/18 (72.2%)	10/11 (90.9%)	9/11 (81.8%)	3/8 (37.5%)
Negative Screen	220/268 (82.1%)	120/149 (80.5%)	100/119 (84.0%)	98/117 (83.8%)	60/75 (80.0%)	46/55 (83.6%)
History of Psychiatric Disorder						
Psychiatric History	110/133 (82.7%)	67/82 (81.7%)	43/51 (84.3%)	57/64 (89.1%)	22/27 (81.5%)	19/27 (70.4%)
No Psychiatric History	160/200 (80.0%)	77/97 (79.4%)	83/103 (80.6%)	62/76 (81.6%)	50/63 (79.4%)	34/43 (79.1%)
Illicit Drug Use						
Drug Use	12/20 (60.0%)	5/8 (62.5%)	7/12 (58.3%)	8/14 (57.1%)	2/2 (100%)	2/4 (50.0%)
No Drug Use	252/306 (82.4%)	137/169 (81.1%)	115/137 (83.9%)	108/123 (87.8%)	69/87 (79.3%)	50/62 (80.6%)
Peptic Ulcer Disease (PUD)						
PUD	19/21 (90.5%)	10/11 (90.9%)	9/10 (90.0%)	9/10 (90.0%)	7/8 (87.5%)	3/3 (100%)
No PUD	250/311 (80.4%)	134/168 (79.8%)	116/143 (81.1%)	110/130 (84.6%)	65/82 (79.3%)	50/67 (74.6%)
Liver Disease						
Liver Disease	7/8 (87.5%)	3/3 (100%)	4/5 (80.0%)	2/3 (66.7%)	3/3 (100%)	2/2 (100%)
No Liver Disease	262/324 (80.9%)	141/176 (80.1%)	121/148 (81.8%)	117/137 (85.4%)	69/87 (79.3%)	51/68 (75.0%)
Connective Tissue Disease (CTD)						
CTD	5/5 (100%)	3/3 (100%)	2/2 (100%)	2/3 (66.7%)	1/1 (100%)	2/2 (100%)
No CTD	264/327 (80.7%)	141/176 (80.1%)	123/151 (81.5%)	117/137 (85.4%)	71/89 (79.8%)	51/68 (75.0%)
Chronic Pulmonary Disease						
Pulmonary Disease	39/46 (84.8%)	23/28 (82.1%)	16/18 (88.9%)	19/22 (86.4%)	7/8 (87.5%)	9/11 (81.8%)
No Pulmonary Disease	230/286 (80.4%)	121/151 (80.1%)	109/135 (80.7%)	100/118 (84.7%)	65/82 (79.3%)	44/59 (74.6%)
Hemiplegia						
Hemiplegia	8/10 (80.0%)	2/3 (66.7%)	6/7 (85.7%)	3/4 (75.0%)	1/1 (100%)	4/5 (80.0%)
No Hemiplegia	261/322 (81.1%)	142/176 (80.7%)	119/146 (81.5%)	116/136 (85.3%)	71/89 (79.8%)	49/65 (75.4%)
Dementia						
Dementia	15/17 (88.2%)	4/4 (100%)	11/13 (84.6%)	7/8 (87.5%)	6/7 (85.7%)	2/2 (100%)
No Dementia	254/315 (80.6%)	140/175 (80.0%)	114/140 (81.4%)	112/132 (84.8%)	66/83 (79.5%)	51/68 (75.0%)
Renal Disease						
Renal Disease	20/21 (95.2%)	13/13 (100%)	7/8 (87.5%)	9/9 (100%)	5/5 (100%)	6/7 (85.7%)
No Renal Disease	249/311 (80.1%)	131/166 (78.9%)	118/145 (81.4%)	110/131 (84.0%)	67/85 (78.8%)	47/63 (74.6%)
Solid Tumor						
Tumor	30/40 (75.0%)	23/27 (85.2%)	7/13 (53.8%)	16/19 (84.2%)	8/9 (88.9%)	3/8 (37.5%)
No Tumor	239/292 (81.8%)	121/152 (79.6%)	118/140 (84.3%)	103/121 (85.1%)	64/81 (79.0%)	50/62 (80.6%)
Autoimmune Disease						
Autoimmune Disease	19/22 (86.4%)	14/16 (87.5%)	5/6 (83.3%)	9/11 (81.8%)	5/5 (100%)	3/4 (75.0%)
No Autoimmune Disease	250/310 (80.6%)	130/163 (79.8%)	120/147 (81.6%)	110/129 (85.3%)	67/85 (78.8%)	50/66 (75.8%)
Thyroid Disease						
Thyroid Disease	33/39 (84.6%)	25/31 (80.6%)	8/8 (100%)	22/25 (88.0%)	6/9 (66.7%)	4/4 (100%)
No Thyroid Disease	236/293 (80.5%)	119/148 (80.4%)	117/145 (80.7%)	97/115 (84.3%)	66/81 (81.5%)	49/66 (74.2%)
Musculoskeletal Disorder (MSK)						
MSK	159/186 (85.5%)	85/99 (85.9%)	74/87 (85.1%)	79/86 (91.9%)	37/46 (80.4%)	30/39 (76.9%)
No MSK	113/150 (75.3%)	60/82 (73.2%)	53/68 (77.9%)	41/55 (74.5%)	36/45 (80.0%)	23/32 (71.9%)
Charlson Comorbidity Index (10-Year Survival Estimate)	292/359 (81.3%)	158/195 (81.0%)	134/164 (81.7%)	128/149 (85.9%)	79/97 (81.4%)	58/78 (74.4%)

**Table 2:** Survey question responses amongst the neurological patient cohort and stratified by sex: crude odds ratios.

	All Participants		Female Participants		Male Participants	
	Odds Ratio (95% CI)	Chi-Square/Fisher Exact Test	Odds Ratio (95% CI)	Chi-Square/Fisher Exact Test	Odds Ratio (95% CI)	Chi-Square/Fisher Exact Test
Q1: Have you had a one-on-one discussion with a physician about the risks and benefits of receiving the COVID vaccination?						
Had Conversation	1.36 (0.67, 2.97)	*p* = 0.46	2.46 (0.87, 8.61)	*p* = 0.11	0.68 (0.24, 2.11)	*p* = 0.60
No Conversation	0.73 (0.34, 1.49)		0.51 (0.15, 1.44)		1.47 (0.47, 4.09)	
Q2: What is your primary source of COVID information?						
Scholarly Articles/CDC/US Governmental Agencies	1.30 (0.61, 3.05)	*p* = 0.60	1.21 (0.46, 3.55)	*p* = 0.85	1.56 (0.42, 8.78)	*p* = 0.77
Friends/Family/Coworkers	0.58 (0.27, 1.32)	*p* = 0.20	0.97 (0.32, 3.56)	*p* = 1.00	0.33 (0.11, 1.09)	*p* = 0.057
Healthcare Provider	1.46 (0.32, 13.69)	*p* = 1.00	0.81 (0.14, 8.28)	*p* = 0.68	2.64 (0.38, 115.04)	*p* = 0.48
Traditional Media	1.82 (1.02, 3.28)	*p* = 0.042	1.40 (0.64, 3.13)	*p* = 0.47	2.51 (1.02, 6.35)	*p* = 0.044
Social Media	0.26 (0.11, 0.63)	*p* = 0.001	0.35 (0.10, 1.26)	*p* = 0.097	0.18 (0.043, 0.72)	*p* = 0.007
Q3: Do you believe that vaccines are safe?						
Safe	11.44 (5.20, 26.09)	*p* < 0.001	11.54 (3.82, 38.07)	*p* < 0.001	11.17 (3.44, 39.13)	*p* < 0.001
Not Safe	0.087 (0.038, 0.19)		0.087 (0.026, 0.26)		0.089 (0.026, 0.29)	
Q4: Do you believe that COVID is a severe illness?						
Severe	4.66 (2.03, 10.65)	*p* < 0.001	4.64 (1.52, 14.06)	*p* = 0.003	4.60 (1.16, 17.66)	*p* = 0.017
Not Severe	0.21 (0.094, 0.49)		0.22 (0.071, 0.66)		0.22 (0.057, 0.86)	
Q5: Do you have a preexisting medical condition that you believe will make the vaccine unsafe?						
Preexisting Condition	0.20 (0.11, 0.37)	*p* < 0.001	0.17 (0.070, 0.40)	*p* < 0.001	0.25 (0.092, 0.66)	*p* = 0.002
No Preexisting Condition	5.03 (2.69, 9.46)		5.91 (2.51, 14.22)		4.08 (1.52, 10.83)	
Q6: Have you received the flu vaccine within the last year?						
Received Flu Shot	5.05 (2.78, 9.40)	*p* < 0.001	6.05 (2.63, 14.46)	*p* < 0.001	4.19 (1.70, 10.95)	*p* = 0.001
Did Not Receive Flu Shot	0.20 (0.11, 0.36)		0.17 (0.069, 0.38)		0.24 (0.091, 0.59)	
Q7: Have you tested positive for COVID?						
Tested Positive	0.45 (0.063, 5.04)	*p* = 0.68	0.70 (0.055, 37.83)	*p* = 0.57	0.21 (0.0027, 17.11)	p = 0.33
Denied Positive Test	2.24 (0.20, 15.99)		1.42 (0.026, 18.32)		4.68 (0.058, 374.36)	
Q8: With a single category, how would you define your race/ethnicity?						
White	1.65 (0.90, 3.08)	*p* = 0.11	1.64 (0.74, 3.78)	*p* = 0.26	1.69 (0.65, 4.80)	*p* = 0.34
Black	4.14 (0.64, 173.87)	*p* = 0.23	0.97 (0.094, 48.27)	*p* = 1.00	3.02 (0.44, 130.14)	*p* = 0.48
Asian	0.97 (0.52, 1.89)	*p* = 1.00	0.64 (0.28, 1.51)	*p* = 0.35	1.84 (0.62, 6.67)	*p* = 0.35
Native Hawaiian or Other Pacific Islander	0.56 (0.30, 1.08)	*p* = 0.079	0.87 (0.34, 2.43)	*p* = 0.94	0.34 (0.13, 0.89)	*p* = 0.022
Hispanic	0.56 (0.30, 1.08)	*p* = 0.17	0.46 (0.092, 2.97)	*p* = 0.38	0.29 (0.032, 3.69)	*p* = 0.20
Native American or Alaskan Native	0.38 (0.11, 1.51)	*p* = 1.00	1.96 (0.26, 88.11)	*p* = 1.00	NA	NA
Q9: How would you define your work status?						
Employed	1.31 (0.71, 2.49)	*p* = 0.45	0.91 (0.41, 2.12)	*p* = 0.97	2.18 (0.79, 6.98)	*p* = 0.16
Homemaker	0.68 (0.20, 2.99)	*p* = 0.51	0.62 (0.17, 2.85)	*p* = 0.49	0.46 (0.023, 27.26)	*p* = 0.46
Not Able to Work	0.46 (0.23, 0.93)	*p* = 0.026	0.59 (0.23, 1.70)	*p* = 0.37	0.34 (0.12, 0.999)	*p* = 0.04
Retired	1.36 (0.76, 2.52)	*p* = 0.34	1.87 (0.79, 4.82)	*p* = 0.18	0.98 (0.41, 2.39)	*p* = 1.00
Student	0.92 (0.18, 9.12)	*p* = 1.00	0.58 (0.090, 6.30)	*p* = 0.62	1.38 (0.16, 64.72)	*p* = 1.00
Unemployed	1.25 (0.34, 6.87)	*p* = 1.00	1.93 (0.24, 88.09)	*p* = 1.00	0.90 (0.17, 9.12)	*p* = 1.00
Q10: What is the highest level of education you completed?						
Graduate Degree	3.60 (1.25, 14.19)	*p* = 0.01	6.71 (1.02, 284.34)	*p* = 0.033	2.54 (0.70, 13.99)	*p* = 0.20
High School Degree	0.37 (0.20, 0.71)	*p* = 0.002	0.38 (0.15, 0.97)	*p* = 0.035	0.37 (0.14, 0.95)	*p* = 0.032
Some College	0.88 (0.46, 1.75)	*p* = 0.82	0.70 (0.30, 1.73)	*p* = 0.50	1.23 (0.43, 4.01)	*p* = 0.87
Some High School	0.88 (0.23, 5.01)	*p* = 0.74	3.75 (0.56, 159.74)	*p* = 0.33	0.27 (0.043, 1.98)	*p* = 0.11
Trade School	0.88 (0.17, 8.74)	*p* = 1.00	1.12 (0.12, 54.43)	*p* = 1.00	0.65 (0.050, 35.39)	*p* = 0.55
Associate/Bachelor’s Degree	1.55 (0.80, 3.13)	*p* = 0.22	1.24 (0.54, 3.03)	*p* = 0.73	2.33 (0.73, 9.86)	*p* = 0.73
Q11: What is your marital status?						
Divorced	0.87 (0.40, 2.08)	*p* = 0.87	0.58 (0.20, 1.97)	*p* = 0.44	1.32 (0.40, 5.70)	*p* = 0.79
Married	1.18 (0.67, 2.09)	*p* = 0.64	1.11 (0.51, 2.43)	*p* = 0.92	1.26 (0.52, 3.07)	*p* = 0.71
Single	0.73 (0.39, 1.41)	*p* = 0.38	0.89 (0.37, 2.24)	*p* = 0.93	0.57 (0.22, 1.60)	*p* = 0.32
Widowed	1.65 (0.55, 6.73)	*p* = 0.48	1.83 (0.50, 10.09)	*p* = 0.42	1.33 (0.15, 63.48)	*p* = 1.00
Q12: How would you describe your political view?						
Conservative	0.63 (0.32, 1.30)	*p* = 0.22	0.50 (0.19, 1.35)	*p* = 0.18	1.09 (0.40, 3.30)	*p* = 1.00
Independent	0.78 (0.41, 1.51)	*p* = 0.52	0.61 (0.24, 1.50)	*p* = 0.32	1.44 (0.57, 3.89)	*p* = 0.53
Liberal	2.20 (1.02, 5.18)	*p* = 0.048	4.10 (1.30, 17.18)	*p* = 0.009	1.49 (0.53, 4.84)	*p* = 0.56

**Table 3:** Survey question responses stratified by race: crude odds ratios.

	White Patients		Asian Patients		NHPI Patients	
	Odds Ratio (95% CI)	Chi-Square or Fisher Exact Test	Odds Ratio (95% CI)	Chi-Square or Fisher Exact Test	Odds Ratio (95% CI)	Chi-Square or Fisher Exact Test
Q1: Have you had a one-on-one discussion with a physician about the risks and benefits of receiving the COVID vaccination?						
Had Conversation	1.83 (0.48, 10.36)	*p* = 0.50	1.47 (0.36, 8.79)	*p* = 0.76	2.09 (0.39, 21.23)	*p* = 0.50
No Conversation	0.48 (0.047, 2.53)		0.68 (0.11, 2.81)		0.48 (0.047, 2.53)	
Q2: What is your primary source of COVID information?						
Scholarly Articles/CDC/US Governmental Agencies	1.84 (0.48, 10.46)	*p* = 0.41	0.41 (0.053, 4.92)	*p* = 0.29	1.76 (0.32, 18.13)	*p* = 0.72
Friends/Family/Coworkers	0.65 (0.16, 3.94)	*p* = 0.46	0.98 (0.17, 10.22)	*p* = 1.00	0.40 (0.10, 1.64)	*p* = 0.23
Healthcare Provider	0.95 (0.11, 45.97)	*p* = 1.00	0.64 (0.048, 35.67)	*p* = 0.55	2.80 (0.36, 128.07)	*p* = 0.45
Traditional Media	1.29 (0.45, 3.72)	*p* = 0.77	2.36 (0.71, 7.98)	*p* = 0.18	1.80 (0.56, 6.00)	*p* = 0.39
Social Media	0.24 (0.042, 1.67)	*p* = 0.079	0.43 (0.099, 2.19)	*p* = 0.24	0.20 (0.015, 1.86)	*p* = 0.093
Q3: Do you believe that vaccines are safe?						
Safe	10.37 (2.57, 43.85)	*p* < 0.001	14.88 (2.15, 172.80)	*p* = 0.002	8.81 (2.14, 41.37)	*p* = 0.001
Not Safe	0.096 (0.023, 0.39)		0.067 (0.0058, 0.46)		0.11 (0.024, 0.47)	
Q4: Do you believe that COVID is a severe illness?						
Severe	11.92 (2.51, 64.47)	*p* = 0.001	0.64 (0.013, 5.56)	*p* = 1.00	4.35 (0.95, 21.01)	*p* = 0.050
Not Severe	0.084 (0.016, 0.40)		1.57 (0.18, 75.46)		0.23 (0.048, 1.05)	
Q5: Do you have a preexisting medical condition that you believe will make the vaccine unsafe?						
Preexisting Condition	0.11 (0.032, 0.34)	*p* < 0.001	0.13 (0.034, 0.45)	*p* < 0.001	0.52 (0.15, 1.80)	*p* = 0.36
No Preexisting Condition	9.23 (2.90, 31.02)		7.87 (2.22, 29.61)		1.92 (0.56, 6.50)	
Q6: Have you received the flu vaccine within the last year?						
Received Flu Shot	7.96 (2.55, 29.90)	*p* < 0.001	5.02 (1.48, 18.16)	*p* = 0.006	2.38 (0.74, 7.75)	*p* = 0.16
Did Not Receive Flu Shot	0.13 (0.033, 0.39)		0.20 (0.055, 0.68)		0.42 (0.13, 1.35)	
Q7: Have you tested positive for COVID?						
Tested Positive	0.079 (0.0013, 1.56)	*p* = 0.053	0.23 (0.0028, 18.34)	*p* = 0.34	1.43 (0.14, 72.07)	*p* = 1.00
Denied Positive Test	12.61 (0.64, 753.84)		4.43 (0.055, 358.89)		0.70 (0.014, 7.37)	
Q9: How would you define your work status?						
Employed	1.14 (0.38, 3.84)	*p* = 1.00	1.14 (0.35, 4.13)	*p* = 1.00	2.09 (0.57, 9.73)	*p* = 0.27
Homemaker	0.81 (0.15, 8.13)	*p* = 0.68	0.23 (0.0028, 18.58)	*p* = 0.34	0.70 (0.036, 42.42)	*p* = 1.00
Not Able to Work	0.46 (0.14, 1.83)	*p* = 0.30	0.28 (0.042, 2.10)	*p* = 0.12	0.58 (0.18, 1.94)	*p* = 0.45
Retired	1.46 (0.51, 4.56)	*p* = 0.60	1.52 (0.48, 5.16)	*p* = 0.59	0.82 (0.24, 3.07)	*p* = 0.94
Student	0.33 (0.017, 19.97)	*p* = 0.37	1.38 (0.16, 64.72)	*p* = 1.00	0.68 (0.034, 42.12)	*p* = 1.00
Unemployed	1.16 (0.14, 54.72)	*p* = 1.00	1.46 (0.17, 68.95)	*p* = 1.00	1.40 (0.13, 72.89)	*p* = 1.00
Q10: What is the highest level of education you completed?						
Graduate Degree	7.09 (1.05, 305.07)	*p* = 0.046	1.23 (0.23, 12.56)	*p* = 1.00	1.38 (0.13, 69.80)	*p* = 1.00
High School Degree	0.14 (0.042, 0.50)	*p* < 0.001	0.62 (0.17, 2.59)	*p* = 0.52	1.00 (0.30, 3.49)	*p* = 1.00
Some College	0.88 (0.27, 3.36)	*p* = 1.00	2.56 (0.52, 25.08)	*p* = 0.34	0.33 (0.094, 1.20)	*p* = 0.092
Some High School	0.46 (0.035, 25.19)	*p* = 0.44	1.83 (0.23, 83.89)	*p* = 1.00	1.35 (0.12, 70.55)	*p* = 1.00
Trade School	1.62 (0.22, 72.29)	*p* = 1.00	0.43 (0.021, 26.38)	*p* = 0.45	0.33 (0.0040, 26.62)	*p* = 0.44
Associate/Bachelor’s Degree	1.62 (0.52, 6.08)	*p* = 0.52	0.68 (0.20, 2.36)	*p* = 0.66	3.87 (0.78, 38.08)	*p* = 0.081
Q11: What is your marital status?						
Divorced	0.75 (0.21, 3.39)	*p* = 0.74	0.42 (0.078, 2.85)	*p* = 0.36	1.18 (0.26, 7.45)	*p* = 1.00
Married	1.85 (0.66, 5.37)	*p* = 0.28	0.96 (0.29, 3.02)	*p* = 1.00	0.86 (0.28, 2.76)	*p* = 0.98
Single	0.49 (0.16, 1.60)	*p* = 0.26	1.47 (0.36, 8.79)	*p* = 0.76	0.91 (0.28, 3.14)	*p* = 1.00
Widowed	1.52 (0.19, 70.14)	*p* = 1.00	1.43 (0.27, 14.40)	*p* = 1.00	1.78 (0.18, 89.14)	*p* = 1.00
Q12: How would you describe your political view?						
Conservative	0.62 (0.19, 2.23)	*p* = 0.56	0.42 (0.091, 1.86)	*p* = 0.31	0.36 (0.065, 2.08)	*p* = 0.30
Independent	0.69 (0.22, 2.20)	*p* = 0.64	1.00 (0.23, 5.15)	*p* = 1.00	0.78 (0.21, 2.80)	*p* = 0.89
Liberal	2.72 (0.70, 15.57)	*p* = 0.17	4.29 (0.54, 197.79)	*p* = 0.27	2.80 (0.64, 17.33)	*p* = 0.22

**Table 4:** Sociodemographic variables for all patients with neurological diseases and stratified by sex: crude odds ratios.

	All Participants		Female Participants		Male Participants	
	Median (IQR)	Wilcoxon Rank Sum Test	Median (IQR)	Wilcoxon Rank Sum Test	Median (IQR)	Wilcoxon Rank Sum Test
Age						
Vaccine Acceptance	61.50 (47.00, 72.00)	7.00 (95% CI: 3.00, 12.00) *p* = 0.003	59.00 (45.00, 70.75)	10.00 (95% CI: 3.00, 17.00) *p* = 0.005	64.00 (52.25, 73.00)	4.00 (95% CI: −2.00, 11.00) *p* = 0.18
Vaccine Declination	55.00 (39.00, 65.00)		46.00 (34.75, 62.50)		61.00 (46.00, 67.50)	
Median Household Income						
Vaccine Acceptance	96,297 (79,074, 102,242)	0.00 (95% CI: −3036, 5661) *p* = 0.93	102,228 (79,506, 103,702)	0.00 (95% CI: −5778 to 8697) *p* = 0.84	93,034 (77,275, 102,242)	0.00 (95% CI: −8697, 9208) *p* = 0.97
Vaccine Declination	93,034 (75,396, 110,939)		94,541 (79,290, 110,939)		92,678 (67,466, 110,939)	
Overall Poverty Level in Municipality						
Vaccine Acceptance	0.056 (0.056, 0.096)	0.00 (95% CI: −0.01, 0.01) *p* = 0.92	0.056 (0.055, 0.091)	0.00 (95% CI: −0.01, 0.01) *p* = 0.59	0.056 (0.056, 0.096)	0.00 (95% CI: −0.015, 0.0070) *p* = 0.66
Vaccine Declination	0.060 (0.049, 0.10)		0.056 (0.049, 0.10)		0.077 (0.049, 0.11)	
Poverty Level for Ages 18–64						
Vaccine Acceptance	0.059 (0.049, 0.090)	0.00 (95% CI: −0.0040, 0.0070) *p* = 0.68	0.059 (0.049, 0.089)	0.00 (95% CI: −0.01, 0.01) *p* = 0.60	0.059 (0.049, 0.091)	0.00 (95% CI: 0.00, 0.01) *p* = 0.97
Vaccine Declination	0.059 (0.049, 0.089)		0.059 (0.049, 0.093)		0.065 (0.049, 0.089)	
Poverty Level for Ages 65 and Older						
Vaccine Acceptance	0.048 (0.042, 0.081)	0.00 (95% CI: −0.001, 0.01) *p* = 0.57	0.043 (0.042, 0.080)	0.00 (95% CI: −0.004, 0.008) *p* = 0.58	0.057 (0.043, 0.081)	0.00 (95% CI: −0.018, 0.0040) *p* = 0.81
Vaccine Declination	0.051 (0.039, 0.093)		0.051 (0.039, 0.088)		0.050 (0.039, 0.11)	
Geographic Origin Population Size						
Vaccine Acceptance	51,511 (28,737, 51,601)	90.00 (95% CI: 0.00, 1974.00) *p* = 0.11	51,511 (28,902, 51,601)	90.00 (95% CI: 0.00, 3262.00) *p* = 0.15	50,741 (27,914, 51,601)	90.00 (95% CI: −1677, 4633) *p* = 0.46
Vaccine Declination	51,511 (33,084, 51,601)		51,511 (41,463, 51,601)		51,556 (25,307, 51,601)	
	**Odds Ratio** **(95% CI)**	**Chi-Square or Fisher Exact Test**	**Odds Ratio** **(95% CI)**	**Chi-Square or Fisher Exact Test**	**Odds Ratio** **(95% CI)**	**Chi-Square or** **Fisher Exact Test**
Insurance Type						
Medicare	1.66 (0.85, 3.42)	*p* = 0.16	13.96 (4.86, 55.21)	*p* < 0.001	1.05 (0.42, 2.73)	*p* = 1.00
Medicaid	0.42 (0.22, 0.82)	*p* = 0.007	0.50 (0.20, 1.31)	*p* = 0.16	0.35 (0.14, 0.94)	*p* = 0.029
Private	1.05 (0.59, 1.90)	*p* = 0.97	0.88 (0.40, 1.93)	*p* = 0.87	1.39 (0.54, 3.91)	*p* = 0.61
Military	1.56 (0.62, 4.73)	*p* = 0.44	0.85 (0.28, 3.14)	*p* = 0.98	5.19 (0.76, 223.73)	*p* = 0.13
Income Quartiles						
Quartile 1	0.95 (0.50, 1.87)	*p* = 0.99	0.95 (0.37, 2.63)	*p* = 1.00	0.94 (0.37, 2.56)	*p* = 1.00
Quartile 2	0.86 (0.46, 1.68)	*p* = 0.74	0.85 (0.35, 2.15)	*p* = 0.85	0.88 (0.34, 2.52)	*p* = 0.97
Quartile 3	2.31 (1.10, 5.33)	*p* = 0.029	2.30 (0.87, 7.21)	*p* = 0.11	2.33 (0.73, 9.86)	*p* = 0.16
Quartile 4	0.59 (0.32, 1.13)	*p* = 0.11	0.59 (0.25, 1.42)	*p* = 0.26	0.60 (0.23, 1.67)	*p* = 0.37
Geographic Origin						
Urban	0.86 (0.48, 1.54)	*p* = 0.70	1.02 (0.46, 2.26)	*p* = 1.00	0.77 (0.31, 1.83)	*p* = 0.65
Suburban	1.18 (0.66, 2.12)	*p* = 0.65	0.98 (0.44, 2.22)	*p* = 1.00	1.03 (0.46, 2.33)	*p* = 1.00
Rural	0.57 (0.091, 6.10)	*p* = 0.62	0.93 (0.088, 46.95)	*p* = 1.00	0.22 (0.003, 17.97)	*p* = 0.34
Sex						
Female	0.96 (0.54, 1.69)	*p* = 0.98				
Male	1.05 (0.59, 1.85)					

**Table 5:** Sociodemographic variables stratified by race: crude odds ratios.

	White Patients		Asian Patients		NHPI Patients	
	Median (25% IQR)	Wilcoxon Rank Sum Test	Median (IQR)	Wilcoxon Rank Sum Test	Median (IQR)	Wilcoxon Rank Sum Test
Age						
Vaccine Acceptance	63.00 (51.00, 72.00)	9.00 (95% CI: 1.00, 17.00) *p* = 0.040	64.00 (46.50, 76.50)	5.00 (95% CI: −5.00, 14.00) *p* = 0.32	56.00 (46.25, 68.00)	8.00 (95% CI: −3.00, 18.00) *p* = 0.16
Vaccine Declination	55.00 (40.00, 64.00)		61.50 (43.75, 67.75)		46.00 (31.50, 62.75)	
Median Household Income						
Vaccine Acceptance	102,242 (79,074, 102,242)	0.00 (95% CI: −5661, 8697) *p* = 0.54	98,384 (79,219, 104,431)	0.00 (95% CI: −9208, 8697) *p* = 0.99	92,321 (81,727, 102,242)	0.00 (95% CI: −8697, 11,916) *p* = 0.81
Vaccine Declination	102,228 (79,506, 110,939)		93,433 (80,172, 110,939)		92,678 (64,866, 110,939)	
Overall Poverty Level in Municipality						
Vaccine Acceptance	0.056 (0.056, 0.089)	0.00 (95% CI: −0.0030, 0.010) *p* = 0.42	0.056 (0.049, 0.086)	0.00 (95% CI: −0.010, 0.011) *p* = 0.59	0.077 (0.056, 0.10)	0.00 (95% CI: −0.0070, 0.028) *p* = 0.61
Vaccine Declination	0.056 (0.049, 0.089)		0.053 (0.049, 0.088)		0.083 (0.049, 0.12)	
Poverty Level for Ages 18–64						
Vaccine Acceptance	0.059 (0.058, 0.091)	0.001 (95% CI: −0.004, 0.01) *p* = 0.27	0.059 (0.049, 0.088)	0.001 (95% CI: −0.006, 0.02) *p* = 0.45	0.066 (0.049, 0.091)	0.003 (95% CI: −0.01, 0.022) *p* = 0.49
Vaccine Declination	0.059 (0.049, 0.085)		0.050 (0.049, 0.078)		0.075 (0.049, 0.11)	
Poverty Level for Ages 65 and Older						
Vaccine Acceptance	0.043 (0.043, 0.071)	0.00 (95% CI: −0.003, 0.004) *p* = 0.56	0.047 (0.039, 0.074)	0.003 (95% CI: −0.004, 0.024) *p* = 0.49	0.057 (0.042, 0.083)	0.00 (95% CI: −0.004, 0.033) *p* = 0.62
Vaccine Declination	0.043 (0.039, 0.079)		0.054 (0.039, 0.093)		0.072 (0.039, 0.10)	
Geographic Origin Population Size						
Vaccine Acceptance	51,511 (25,307, 51,511)	90.00 (95% CI: −1677, 3163) *p* = 0.47	51,511 (46,690, 51601)	1470 (95% CI: 0.00, 5999.00) *p* = 0.14	49,971 (14,856, 516,01)	1630 (95% CI: −90.00, 19,079.00) *p* = 0.17
Vaccine Declination	49,834 (42,069, 51,601)		51,601 (43,101, 55479)		51,601 (29,899, 51,946)	
	**Odds Ratio** **(95% CI)**	**Chi-Square or Fisher Exact Test**	**Odds Ratio** **(95% CI)**	**Chi-Square or Fisher Exact Test**	**Odds Ratio** **(95% CI)**	**Chi-Square or Fisher Exact Test**
Insurance Type						
Medicare	1.69 (0.54, 6.30)	*p* = 0.47	1.85 (0.51, 8.49)	*p* = 0.41	1.18 (0.30, 5.70)	*p* = 1.00
Medicaid	0.34 (0.10, 1.23)	*p* = 0.089	1.15 (0.27, 7.00)	*p* = 1.00	0.48 (0.15, 1.58)	*p* = 0.27
Private	0.71 (0.25, 2.06)	*p* = 0.63	0.88 (0.27, 2.80)	*p* = 1.00	1.81 (0.55, 6.60)	*p* = 0.41
Military	4.63 (0.67, 200.92)	*p* = 0.20	0.055 (0.0011, 0.57)	*p* = 0.005	1.05 (0.079, 58.28)	*p* = 1.00
Income Quartiles						
Quartile 1	1.95 (0.52, 11.02)	*p* = 0.41	1.13 (0.30, 5.26)	*p* = 1.00	0.69 (0.20, 2.65)	*p* = 0.73
Quartile 2	0.59 (0.19, 2.06)	*p* = 0.48	0.60 (0.18, 2.25)	*p* = 0.55	1.46 (0.44, 5.35)	*p* = 0.68
Quartile 3	2.35 (0.71, 10.18)	*p* = 0.21	3.11 (0.64, 30.15)	*p* = 0.23	1.68 (0.30, 17.43)	*p* = 0.72
Quartile 4	0.43 (0.14, 1.32)	*p* = 0.14	0.68 (0.20, 2.53)	*p* = 0.70	0.69 (0.20, 2.65)	*p* = 0.73
Geographic Origin						
Urban	1.21 (0.43, 3.42)	*p* = 0.87	0.76 (0.21, 2.47)	*p* = 0.81	0.65 (0.20, 2.03)	*p* = 0.56
Suburban	0.91 (0.32, 2.58)	*p* = 1.00	1.32 (0.40, 4.74)		1.44 (0.46, 4.73)	*p* = 0.66
Rural	0.49 (0.037, 26.87)	*p* = 0.46	NA	NA	0.72 (0.04, 43.18)	*p* = 1.00
Sex						
Female	0.82 (0.27, 2.30)	*p* = 0.86	0.40 (0.10, 1.33)	*p* = 0.16	1.60 (0.51, 5.24)	*p* = 0.52
Male	1.22 (0.43, 3.65)		2.51 (0.75, 9.88)		0.63 (0.19, 1.96)	

**Table 6:** Crude odds of vaccination by medical comorbidity for all patients with neurological disorders and stratified by sex.

	All Participants		Female Participants		Male Patients	
	Odds Ratio (95% CI)	Chi-Square or Fisher Exact Test	Odds Ratio (95% CI)	Chi-Square or Fisher Exact Test	Odds Ratio (95% CI)	Chi-Square or Fisher Exact Test
Dyslipidemia						
Dyslipidemia	2.02 (1.11, 3.76)	*p* = 0.021	2.18 (0.91, 5.67)	*p* = 0.091	1.91 (0.77, 4.78)	*p* = 0.18
No Dyslipidemia	0.50 (0.27, 0.90)		0.46 (0.18, 1.10)		0.52 (0.21, 1.30)	
Type 1 or 2 Diabetes Mellitus						
Diabetes Mellitus	1.17 (0.52, 2.90)	*p* = 0.83	1.92 (0.53, 10.63)	*p* = 0.42	0.78 (0.27, 2.64)	*p* = 0.84
No Diabetes Mellitus	0.85 (0.34, 1.91)		0.52 (0.094, 1.90)		1.27 (0.38, 3.76)	
Hypertension						
Hypertension	1.60 (0.88, 2.96)	*p* = 0.13	1.94 (0.83, 4.87)	*p* = 0.15	1.29 (0.52, 3.21)	*p* = 0.69
No Hypertension	0.62 (0.34, 1.14)		0.52 (0.21, 1.21)		0.78 (0.31, 1.91)	
Coronary Artery Disease or Prior Myocardial Infarction (CAD/MI)						
CAD/MI	0.71 (0.29, 1.91)	*p* = 0.56	0.88 (0.22, 5.21)	*p* = 0.74	0.58 (0.18, 2.27)	*p* = 0.52
No CAD/MI	1.42 (0.53, 3.46)		1.13 (0.19, 4.64)		1.72 (0.44, 5.71)	
Peripheral Vascular Disease (PVD)						
PVD	0.77 (0.19, 4.50)	*p* = 0.72	3.04 (0.44, 131.87)	*p* = 0.48	0.28 (0.044, 2.02)	*p* = 0.12
No PVD	1.29 (0.22, 5.23)		0.33 (0.0076, 2.30)		3.59 (0.50, 22.67)	
Smoking Status						
Current Smoker	0.50 (0.21, 1.26)	*p* = 0.14	0.49 (0.16, 1.69)	*p* = 0.28	0.53 (0.14, 2.53)	*p* = 0.29
Former Smoker	1.25 (0.56, 3.10)	*p* = 0.70	5.50 (0.82, 235.56)	*p* = 0.081	0.67 (0.25, 1.97)	*p* = 0.55
Never Smoker	1.23 (0.63, 2.33)	*p* = 0.60	0.83 (0.28, 2.19)	*p* = 0.87	1.80 (0.70, 4.54)	*p* = 0.24
Congestive Heart Failure (CHF)						
CHF	1.42 (0.17, 66.33)	*p* = 1.00	1.49 (0.18, 69.45)	*p* = 1.00	0.67 (0.052, 36.43)	*p* = 0.56
No CHF	0.70 (0.015, 5.97)		0.67 (0.014, 5.68)		1.49 (0.027, 19.38)	
Atrial Fibrillation (Afib)						
Afib	1.69 (0.48, 9.14)	*p* = 0.59	2.26 (0.29, 102.19)	*p* = 0.69	1.38 (0.28, 13.42)	*p* = 1.00
No Afib	0.59 (0.11, 2.08)		0.44 (0.0098, 3.39)		0.73 (0.075, 3.57)	
Cerebrovascular Accident (CVA)						
CVA	2.33 (0.87, 7.85)	*p* = 0.12	6.42 (0.97, 273.63)	*p* = 0.051	1.28 (0.38, 5.58)	*p* = 0.79
No CVA	0.43 (0.13, 1.15)		0.16 (0.0037, 1.03)		0.78 (0.18, 2.61)	
Alcohol Use Screen						
Positive Screen	0.97 (0.41, 2.57)	*p* = 1.00	0.56 (0.19, 1.93)	*p* = 0.41	2.22 (0.48, 20.93)	*p* = 0.37
Negative Screen	1.03 (0.39, 2.44)		1.77 (0.52, 5.37)		0.45 (0.048, 2.08)	
Alcohol Use Disorder						
Alcohol Use Disorder	1.69 (0.20, 77.30)	*p* = 1.00	0.12 (0.00, 2.35)	*p* = 0.10	3.40 (0.50, 146.59)	*p* = 0.32
No Alcohol Use Disorder	0.59 (0.013, 4.75)		8.28 (0.43, 492.80)		0.29 (0.0068, 2.01)	
Depression Screen						
Positive Screen	0.68 (0.28, 1.86)	*p* = 0.52	0.97 (0.24, 5.68)	*p* = 1.00	0.50 (0.14, 1.99)	*p* = 0.37
Negative Screen	1.46 (0.54, 3.61)		1.03 (0.18, 4.19)		2.01 (0.50, 6.96)	
History of Psychiatric Disorder						
Psychiatric History	1.20 (0.66, 2.22)	*p* = 0.63	1.16 (0.52, 2.64)	*p* = 0.84	1.29 (0.49, 3.68)	*p* = 0.73
No Psychiatric History	0.84 (0.45, 1.52)		0.86 (0.38, 1.93)		0.77 (0.27, 2.02)	
Illicit Drug Use						
Drug Use	0.32 (0.11, 0.96)	*p* = 0.030	0.39 (0.072, 2.65)	*p* = 0.19	0.27 (0.067, 1.19)	*p* = 0.069
No Drug Use	3.10 (1.04, 8.71)		2.55 (0.38, 13.91)		3.69 (0.84, 14.97)	
Peptic Ulcer Disease (PUD)						
PUD	2.31 (0.53, 21.02)	*p* = 0.39	2.53 (0.34, 113.26)	*p* = 0.69	2.09 (0.27, 95.14)	*p* = 0.78
No PUD	0.43 (0.048, 1.87)		0.40 (0.0088, 2.96)		0.48 (0.011, 3.72)	
Liver Disease						
Liver Disease	1.65 (0.21, 75.82)	*p* = 1.00	1.49 (0.18, 69.45)	*p* = 1.00	0.89 (0.084, 45.56)	*p* = 1.00
No Liver Disease	0.60 (0.013, 4.85)		0.67 (0.014, 5.68)		1.12 (0.022, 11.91)	
Connective Tissue Disease (CTD)						
CTD	2.38 (0.33, 104.33)	*p* = 0.70	1.49 (0.18, 69.45)	*p* = 1.00	0.91 (0.088, 45.63)	*p* = 1.00
No CTD	0.42 (0.0096, 3.00)		0.67 (0.014, 5.68)		1.10 (0.022, 11.38)	
Chronic Pulmonary Disease						
Pulmonary Disease	1.36 (0.56, 3.78)	*p* = 0.62	1.14 (0.38, 4.16)	*p* = 1.00	1.90 (0.41, 18.08)	*p* = 0.53
No Pulmonary Disease	0.74 (0.26, 1.79)		0.88 (0.24, 2.63)		0.53 (0.055, 2.47)	
Hemiplegia						
Hemiplegia	0.94 (0.18, 9.26)	*p* = 1.00	0.48 (0.024, 29.07)	*p* = 0.48	1.36 (0.15, 64.89)	*p* = 0.48
No Hemiplegia	1.07 (0.11, 5.55)		2.08 (0.034, 41.00)		0.74 (0.015, 6.46)	
Dementia						
Dementia	1.80 (0.40, 16.63)	*p* = 0.75	2.00 (0.26, 89.97)	*p* = 1.00	1.25 (0.25, 12.31)	*p* = 1.00
No Dementia	0.56 (0.060, 2.49)		0.50 (0.011, 3.84)		0.80 (0.081, 4.00)	
Renal Disease						
Renal Disease	4.97 (0.76, 209.52)	*p* = 0.14	6.93 (1.10, 288.47)	*p* = 0.075	1.60 (0.19, 74.80)	*p* = 1.00
No Renal Disease	0.20 (0.004, 1.31)		0.14 (0.004, 0.91)		0.63 (0.013, 5.21)	
Solid Tumor						
Tumor	0.67 (0.29, 1.62)	*p* = 0.41	1.47 (0.45, 6.28)	*p* = 0.68	0.22 (0.057, 0.87)	*p* = 0.019
No Tumor	1.50 (0.62, 3.40)		0.68 (0.16, 2.20)		4.53 (1.14, 17.51)	
Autoimmune Disease						
Autoimmune Disease	1.52 (0.43, 8.27)	*p* = 0.78	1.77 (0.38, 16.83)	*p* = 0.68	1.12 (0.12, 55.16)	*p* = 1.00
No Autoimmune Disease	0.66 (0.12, 2.35)		0.56 (0.059, 2.65)		0.89 (0.018, 8.43)	
Thyroid Disease						
Thyroid Disease	1.33 (0.52, 4.06)	*p* = 0.70	1.02 (0.36, 3.31)	*p* = 1.00	3.82 (0.57, 163.30)	*p* = 0.21
No Thyroid Disease	0.75 (0.25, 1.94)		0.98 (0.30, 2.77)		0.26 (0.006, 1.76)	
Musculoskeletal Disorder (MSK)						
MSK	1.92 (1.07, 3.49)	*p* = 0.027	2.22 (0.99, 5.09)	*p* = 0.052	1.61 (0.65, 4.01)	*p* = 0.35
No MSK	0.52 (0.29, 0.93)		0.45 (0.20, 1.01)		0.62 (0.25, 1.53)	
	**Median** **(IQR)**	**Wilcoxon Rank Sum Test**	**Median** **(IQR)**	**Wilcoxon Rank Sum Test**	**Median** **(IQR)**	**Wilcoxon Rank Sum Test**
Body Mass Index						
Vaccine Acceptance	27.02 (23.20, 32.02)	1.09 (95% CI: −0.66, 2.79) *p* = 0.22	25.82 (21.97, 30.99)	0.48 (95% CI: −1.72, 2.45) *p* = 0.64	28.46 (24.14, 32.55)	2.81 (95% CI: −0.02, 5.76) *p* = 0.052
Vaccine Declination	27.56 (24.39, 33.13)		25.45 (23.64, 31.11)		31.05 (26.56, 36.31)	
Charlson Comorbidity Index (CCI)						
Vaccine Acceptance	3.00 (1.00, 4.00)	1.00 (95% CI: $4.75\times 10^{-5}$, 1.00) *p* = 0.0019	2.00 (1.00, 4.00)	1.00 (95% CI: 1.00 to 2.00) *p* = 0.00021	3.00 (2.00, 4.00)	$9.56\times 10^{-6}\;$(95% CI: −1.00, 1.00) *p* = 0.72
Vaccine Declination	2.00 (0.00, 3.00)		1.00 (0.00, 2.00)		3.00 (0.25, 4.00)	
10-Year Survival Estimate						
Vaccine Acceptance	77.48 (53.39, 95.87)	5.72 (95% CI: 0.00, 8.15) *p* = 0.002	90.15 (53.39, 95.87)	8.15 (95% CI: 2.43, 18.39) *p* < 0.001	77.48 (53.39, 90.15)	0.00 (95% CI: −5.72, 8.15) *p* = 0.72
Vaccine Declination	90.15 (77.48, 98.30)		95.87 (90.15, 98.30)		77.48 (53.39, 97.69)	

**Table 7:** Crude odds of vaccination by medical comorbidity stratified by race.

	White Patients		Asian Patients		NHPI Patients	
	Odds Ratio (95% CI)	Chi-Square or Fisher Exact Test	Odds Ratio (95% CI)	Chi-Square or Fisher Exact Test	Odds Ratio (95% CI)	Chi-Square or Fisher Exact Test
Dyslipidemia						
Dyslipidemia	3.75 (1.13, 16.21)	*p* = 0.018	1.39 (0.43, 4.50)	*p* = 0.71	2.19 (0.63, 8.35)	*p* = 0.26
No Dyslipidemia	0.27 (0.062, 0.88)		0.72 (0.22, 2.31)		0.46 (0.12, 1.59)	
Type 1 or 2 Diabetes Mellitus						
Diabetes Mellitus	0.52 (0.14, 2.46)	*p* = 0.29	5.19 (0.70, 232.08)	*p* = 0.11	1.51 (0.34, 9.45)	*p* = 0.74
No Diabetes Mellitus	1.91 (0.41, 7.20)		0.19 (0.0043, 1.42)		0.66 (0.11, 2.95)	
Hypertension						
Hypertension	3.88 (1.05, 21.71)	*p* = 0.028	1.25 (0.39, 4.01)	*p* = 0.87	1.25 (0.36, 4.31)	*p* = 0.90
No Hypertension	0.26 (0.046, 0.95)		0.80 (0.25, 2.58)		0.80 (0.23, 2.80)	
Coronary Artery Disease or Prior Myocardial Infarction (CAD/MI)						
CAD/MI	0.51 (0.082, 5.50)	*p* = 0.34	1.44 (0.27, 14.64)	*p* = 0.94	0.50 (0.11, 2.70)	*p* = 0.53
No CAD/MI	1.97 (0.18, 12.13)		0.70 (0.068, 3.70)		2.00 (0.37, 9.43)	
Peripheral Vascular Disease (PVD)						
PVD	0.72 (0.069, 36.19)	*p* = 0.57	0.49 (0.024, 30.32)	*p* = 0.49	0.96 (0.15, 10.69)	*p* = 1.00
No PVD	1.39 (0.028, 14.51)		2.04 (0.033, 41.35)		1.04 (0.094, 6.69)	
Smoking Status						
Current Smoker	0.27 (0.069, 1.15)	*p* = 0.061	1.01 (0.092, 52.89)	*p* = 1.00	0.58 (0.13, 3.07)	*p* = 0.47
Former Smoker	0.82 (0.23, 3.71)	*p* = 0.75	1.02 (0.23, 6.32)	*p* = 1.00	1.95 (0.36, 20.10)	*p* = 0.50
Never Smoker	2.39 (0.80, 6.95)	*p* = 0.12	0.98 (0.21, 3.75)	*p* = 1.00	0.98 (0.25, 3.45)	*p* = 1.00
Congestive Heart Failure (CHF)						
CHF	0.72 (0.069, 36.19)	*p* = 0.57	0.25 (0.003, 20.18)	*p* = 0.37	2.03 (0.23, 96.52)	*p* = 0.68
No CHF	1.39 (0.028, 14.51)		4.03 (0.05, 326.77)		0.49 (0.010, 4.29)	
Atrial Fibrillation (Afib)						
Afib	3.43 (0.51, 146.46)	*p* = 0.33	1.00 (0.091, 52.11)	*p* = 1.00	1.14 (0.19, 12.40)	*p* = 1.00
No Afib	0.29 (0.007, 1.95)		1.00 (0.019, 11.00)		0.88 (0.081, 5.33)	
Cerebrovascular Accident (CVA)						
CVA	3.95 (0.48, 143.59)	*p* = 0.077	0.81 (0.18, 5.13)	*p* = 0.72	2.66 (0.51, 26.93)	*p* = 0.32
No CVA	0.00 (0.01, 2.06)		1.24 (0.20, 5.66)		0.38 (0.037, 1.96)	
Alcohol Use Screen						
Positive Screen	0.63 (0.19, 2.46)	*p* = 0.61	0.73 (0.12, 8.05)	*p* = 0.66	2.76 (0.35, 126.66)	*p* = 0.45
Negative Screen	1.59 (0.41, 5.30)		1.37 (0.12, 8.65)		0.36 (0.0079, 2.87)	
Alcohol Use Disorder						
Alcohol Use Disorder	0.90 (0.093, 44.71)	*p* = 1.00	NA	NA	1.33 (0.13, 67.55)	*p* = 1.00
No Alcohol Use Disorder	1.11 (0.022, 10.70)		NA		0.75 (0.015, 7.94)	
Depression Screen						
Positive Screen	1.93 (0.25, 88.48)	*p* = 1.00	1.12 (0.20, 11.76)	*p* = 1.00	0.12 (0.016, 0.76)	*p* = 0.010
Negative Screen	0.52 (0.011, 4.05)		0.89 (0.085, 5.00)		8.11 (1.32, 62.02)	
History of Psychiatric Disorder						
Psychiatric History	1.83 (0.64, 5.76)	*p* = 0.32	1.14 (0.33, 4.60)	*p* = 1.00	0.63 (0.18, 2.23)	*p* = 0.59
No Psychiatric History	0.55 (0.17, 1.57)		0.88 (0.22, 3.04)		1.58 (0.45, 5.51)	
Illicit Drug Use						
Drug Use	0.19 (0.049, 0.75)	*p* = 0.009	1.04 (0.099, 52.80)	*p* = 1.00	3.26 (0.22, 48.59)	*p* = 0.25
No Drug Use	5.30 (1.33, 20.39)		0.96 (0.019, 10.08)		0.31 (0.021, 4.56)	
Peptic Ulcer Disease (PUD)						
PUD	1.63 (0.21, 75.24)	*p* = 1.00	1.82 (0.21, 87.27)	*p* = 1.00	2.03 (0.23, 96.52)	*p* = 0.68
No PUD	0.61 (0.013, 4.87)		0.55 (0.011, 4.78)		0.49 (0.010, 4.29)	
Liver Disease						
Liver Disease	0.35 (0.017, 21.17)	*p* = 0.39	1.56 (0.18, 73.95)	*p* = 1.00	1.33 (0.13, 67.55)	*p* = 1.00
No Liver Disease	2.89 (0.047, 58.09)		0.64 (0.014, 5.54)		0.75 (0.015, 7.94)	
Connective Tissue Disease (CTD)						
CTD	0.72 (0.069, 36.19)	*p* = 0.57	0.51 (0.026, 30.74)	*p* = 0.50	1.33 (0.13, 67.55)	*p* = 1.00
No CTD	1.39 (0.028, 14.51)		1.96 (0.033, 38.66)		0.75 (0.015, 7.94)	
Chronic Pulmonary Disease						
Pulmonary Disease	1.14 (0.29, 6.62)	*p* = 1.00	1.82 (0.21, 87.27)	*p* = 1.00	1.53 (0.27, 16.08)	*p* = 1.00
No Pulmonary Disease	0.88 (0.15, 3.47)		0.55 (0.011, 4.78)		0.66 (0.062, 3.71)	
Hemiplegia						
Hemiplegia	0.52 (0.039, 28.50)	*p* = 0.48	0.51 (0.026, 30.74)	*p* = 0.50	1.30 (0.12, 68.27)	*p* = 1.00
No Hemiplegia	1.92 (0.035, 25.35)		1.96 (0.033, 38.66)		0.77 (0.015, 8.54)	
Dementia						
Dementia	1.25 (0.15, 59.12)	*p* = 1.00	1.54 (0.17, 75.19)	*p* = 1.00	1.33 (0.13, 67.55)	*p* = 1.00
No Dementia	0.80 (0.017, 6.81)		0.65 (0.013, 5.94)		0.75 (0.015, 7.94)	
Renal Disease						
Renal Disease	3.43 (0.51, 146.46)	*p* = 0.33	2.68 (0.36, 119.73)	*p* = 0.47	2.02 (0.22, 99.70)	*p* = 1.00
No Renal Disease	0.29 (0.0068, 1.95)		0.37 (0.0084, 2.79)		0.49 (0.010, 4.58)	
Solid Tumor						
Tumor	0.93 (0.23, 5.49)	*p* = 1.00	2.11 (0.25, 99.72)	*p* = 0.68	0.15 (0.020, 0.89)	*p* = 0.017
No Tumor	1.07 (0.18, 4.33)		0.47 (0.010, 3.97)		6.69 (1.13, 49.16)	
Autoimmune Disease						
Autoimmune Disease	0.78 (0.14, 7.96)	*p* = 0.67	2.68 (0.36, 119.73)	*p* = 0.47	0.96 (0.071, 53.51)	*p* = 1.00
No Autoimmune Disease	1.28 (0.13, 6.93)		0.37 (0.0084, 2.79)		1.04 (0.019, 14.05)	
Thyroid Disease						
Thyroid Disease	1.36 (0.35, 7.82)	*p* = 0.77	0.46 (0.086, 3.16)	*p* = 0.38	2.76 (0.35, 126.66)	*p* = 0.45
No Thyroid Disease	0.74 (0.13, 2.86)		2.18 (0.32, 11.68)		0.36 (0.0079, 2.87)	
Musculoskeletal Disorder (MSK)						
MSK	3.27 (1.09, 10.61)	*p* = 0.031	1.03 (0.32, 3.30)	*p* = 1.00	1.30 (0.39, 4.37)	*p* = 0.83
No MSK	0.26 (0.083, 0.76)		0.97 (0.30, 3.12)		0.77 (0.23, 2.58)	
	**Median (IQR)**	**Wilcoxon Rank Sum Test**	**Median (IQR)**	**Wilcoxon Rank Sum Test**	**Median (IQR)**	**Wilcoxon Rank Sum Test**
Body Mass Index						
Vaccine Acceptance	27.46 (22.86, 31.60)	1.78 (95% CI: −1.08, 4.46) *p* = 0.23	24.66 (22.45, 28.97)	1.52 (95% CI: −0.92, 4.98) *p* = 0.25	31.08 (26.80, 37.37)	3.45 (95% CI: −1.14, 8.37) *p* = 0.17
Vaccine Declination	25.24 (23.71, 27.25)		25.45 (23.41, 32.30)		34.40 (30.93, 42.06)	
Charlson Comorbidity Index						
Vaccine Acceptance	3.00 (2.00, 4.00)	1.00 (95% CI: 0.00, 2.00) *p* = 0.022	3.00 (1.00, 4.00)	1.00 (95% CI: 0.00, 2.00) *p* = 0.25	2.00 (1.00, 4.00)	0.00 (95% CI: 0.00, 2.00) *p* = 0.32
Vaccine Declination	1.00 (0.00, 3.00)		2.00 (1.00, 3.00)		2.00 (0.00, 3.25)	
10-Year Survival Estimate						
Vaccine Acceptance	77.48 (53.39, 90.15)	8.15 (95% CI: 0.00, 20.82) *p* = 0.022	77.48 (53.39, 95.87)	2.43 (95% CI: 0.00, 20.82) *p* = 0.25	90.15 (53.39, 95.87)	0.00 (95% CI: 0.00, 8.15) *p* = 0.32
Vaccine Declination	95.87 (77.48, 98.30)		90.15 (77.48, 95.87)		90.15 (71.46, 98.30)	

**Table 8:** Univariate and multivariable analysis of variables associated with vaccine hesitancy for all patients with neurological disorders and patients stratified by sex.

	Total Patients		Female Patients		Male Patients	
	Unadjusted Odds Ratios (95% CI)	Best Fit Model: Adjusted Odds Ratios	Unadjusted Odds Ratios (95% CI)	Best Fit Model: Adjusted Odds Ratios	Unadjusted Odds Ratios (95% CI)	Best Fit Model: Adjusted Odds Ratios
Age	1.02 (1.01, 1.04), *p* = 0.003	1.02 (0.99, 1.06), *p* = 0.18	1.03 (1.01, 1.05), *p* = 0.006		1.02 (0.99, 1.04), *p* = 0.21	
Median Household Income	1.00 (1.00, 1.00), *p* = 0.56		1.00 (1.00, 1.00), *p* = 0.600		1.00 (1.00, 1.00), *p* = 0.77	
Overall Poverty Level	0.19 (0.00, 44.58), *p* = 0.55		0.30 (0.00, 218.85), *p* = 0.72		0.068 (0.00, 1469.94), *p* = 0.60	
Poverty Level Ages 18–64	0.74 (0.002, 345.46), *p* = 0.92		0.66 (0.00, 956.29), *p* = 0.91		0.95 (0.00, 82118.96), *p* = 0.99	
Poverty Level 65 and Older	0.007 (0.00, 1.70), *p* = 0.077		0.045 (0.00, 49.04), *p* = 0.38		0.00 (0.00, 2.31), *p* = 0.076	
Origin Population Size	1.00 (1.00, 1.00), *p* = 0.29		1.00 (1.00, 1.00), *p* = 0.15		1.00 (1.00, 1.00), *p* = 0.99	
Geographic Origin						
Urban	Referent		Referent		Referent	
Suburban	1.16 (0.67, 2.00), *p* = 0.61		0.98 (0.47, 2.06), *p* = 0.96		1.40 (0.62, 3.15), *p* = 0.42	
Rural	0.60 (0.11, 3.24), *p* = 0.56		0.92 (0.097, 8.68), *p* = 0.94		0.26 (0.015, 4.33), *p* = 0.35	
Income Quartiles						
Third Quartile (Middle Class)	Referent		Referent		Referent	
First Quartile	0.50 (0.21, 1.15), *p* = 0.10		0.51 (0.16, 1.59), *p* = 0.24		0.48 (0.14, 1.71), *p* = 0.26	
Second Quartile	0.46 (0.20, 1.07), *p* = 0.071		0.47 (0.16, 1.39), *p* = 0.17		0.46 (0.13, 1.66), *p* = 0.24	
Fourth Quartile	0.35 (0.15, 0.80), *p* = 0.013		0.36 (0.12, 1.04), *p* = 0.060		0.34 (0.095, 1.23), *p* = 0.10	
Insurance						
Private	Referent		Referent		Referent	
Medicaid	0.50 (0.26, 0.98), *p* = 0.042		0.62 (0.25, 1.52), *p* = 0.29		0.36 (0.13, 1.03), *p* = 0.058	
Medicare	1.42 (0.70, 2.87), *p* = 0.33		2.70 (0.86, 8.52), *p* = 0.090		0.82 (0.30, 2.27), *p* = 0.70	
Military	1.44 (0.55, 3.77), *p* = 0.45		0.93 (0.30, 2.83), *p* = 0.89		3.64 (0.43, 31.06), *p* = 0.24	
Sex						
Female	Referent					
Male	1.05 (0.61, 1.78), *p* = 0.87					
Q1: Have you had a one-on-one discussion with a physician about the risks and benefits of receiving the COVID vaccination?						
Had Conversation	Referent		Referent		Referent	
No Conversation	0.73 (0.37, 1.45), *p* = 0.37		0.40 (0.15, 1.11), *p* = 0.078		0.40 (0.15, 1.11), *p* = 0.078	
Q2: Primary source of COVID information						
Scholarly Articles/ CDC/ US Governmental Agencies	Referent	Referent	Referent		Referent	Referent
Healthcare Provider	1.16 (0.23, 5.95), *p* = 0.86	0.13 (0.01, 1.84), *p* = 0.13	0.70 (0.12, 4.10), *p* = 0.69		7.16 (0.00, $7.33\times 10^{5}$), *p* = 0.99	0.15 (0.00, 254.89), *p* = 1.00
Friends/Family/Coworkers	0.51 (0.20, 1.30), *p* = 0.16	0.55 (0.075, 3.99), *p* = 0.55	0.84 (0.24, 2.99), *p* = 0.79		0.27 (0.058, 1.21), *p* = 0.087	0.10 (0.00, 17.29), *p* = 0.99
Traditional Media (TV News, Radio, Print Media)	1.08 (0.49, 2.36), *p* = 0.85	0.37 (0.077, 1.79), *p* = 0.22	1.03 (0.39, 2.71), *p* = 0.96		1.04 (0.27, 4.00), *p* = 0.96	0.28 (0.002, 35.35), *p* = 0.99
Social Media	0.24 (0.087, 0.65), *p* = 0.005	0.069 (0.01, 0.56), *p* = 0.013	0.33 (0.091, 1.22), *p* = 0.097		0.14 (0.027, 0.75), *p* = 0.021	0.042 (0.00021, 8.43), *p* = 0.99
Q3: Do you believe that vaccines are safe?						
Yes	Referent	Referent	Referent	Referent	Referent	Referent
No	0.086 (0.041, 0.18), *p* < 0.001	0.16 (0.038, 0.71), *p* = 0.015	0.085 (0.031, 0.23), *p* < 0.001	0.081 (0.012, 0.54), *p* = 0.009	0.087 (0.030, 0.26), *p* < 0.001	0.67 (0.059, 7.67), *p* = 0.71
Q4: Do you believe that COVID is a severe illness?						
Yes	Referent	Referent	Referent		Referent	Referent
No	0.21 (0.10, 0.45), *p* < 0.001	0.20 (0.030, 1.25), *p* = 0.085	0.21 (0.079, 0.57), *p* = 0.002		0.21 (0.066, 0.70), *p* = 0.010	0.00 (0.00, 0.47), *p* = 0.037
Q5: Do you have a preexisting medical condition that you believe will make the vaccine unsafe?						
Yes	Referent	Referent	Referent	Referent	Referent	Referent
No	5.06 (2.82, 9.10), *p* < 0.001	10.25 (3.32, 31.69), *p* < 0.001	5.99 (2.71, 13.21), *p* < 0.001	9.21 (2.64, 32.20), *p* = 0.001	4.12 (1.70, 9.99), *p* = 0.002	3.09 (0.33, 28.55), *p* = 0.063
Q6: Have you received the flu vaccine within the last year?						
Yes	Referent	Referent	Referent	Referent	Referent	Referent
No	0.20 (0.11, 0.35), *p* < 0.001	0.067 (0.018, 0.25), *p* < 0.001	0.16 (0.075, 0.36), *p* < 0.001	0.24 (0.067, 0.89), *p* = 0.033	0.24 (0.10, 0.55), *p* = 0.001	0.00 (0.00, 0.60), *p* = 0.037
Q7: Have you tested positive for COVID?						
Yes	Referent		Referent		Referent	
No	2.24 (0.40, 12.51), *p* = 0.36		1.43 (0.14, 14.11), *p* = 0.76		4.75 (0.29, 78.24), *p* = 0.28	
Q8: With a single category, how would you define your race/ethnicity?						
White	Referent		Referent		Referent	Referent
Asian	0.72 (0.36, 1.43), *p* = 0.35		0.55 (0.23, 1.29), *p* = 0.17		1.13 (0.35, 3.73), *p* = 0.84	2.54 (0.12, 55.07), *p* = 0.14
Hispanic	0.30 (0.090, 0.97), *p* = 0.044		0.36 (0.079, 1.61), *p* = 0.18		0.22 (0.031, 1.51), *p* = 0.12	0.31 (0.003, 33.76), *p* = 0.81
Native Hawaiian/Other Pacific Islander	0.48 (0.24, 0.95), *p* = 0.034		0.67 (0.25, 1.78), *p* = 0.42		0.34 (0.12, 0.93), *p* = 0.035	0.46 (0.030, 6.86), *p* = 0.066
Black	8.87 (0.001, $1.07\times 10^{5}$), *p* = 0.99		1.17 (0.00, 3021.67), *p* = 0.99		7.30 (0.00, $2.96\;x\;10^{5})$, *p* = 0.99	1.78 (0.006, 514.63), *p* = 1.00
Native American	2.33 (0.0024, 2306.47), *p* = 0.99		6.89 (0.00, $8.86\times 10^{6}$), *p* = 0.99		NA	NA
Q9: How would you define your work status?						
Employed	Referent		Referent		Referent	
Unemployed	1.02 (0.27, 3.86), *p* = 0.97		2.00 (0.23, 17.44), *p* = 0.53		0.51 (0.087, 2.99), *p* = 0.46	
Homemaker	0.58 (0.17, 1.98), *p* = 0.38		0.69 (0.19, 2.51), *p* = 0.57		0.25 (0.00, 614.51), *p* = 1.00	
Not Able to Work	0.44 (0.21, 0.93), *p* = 0.033		0.69 (0.25, 1.89), *p* = 0.47		0.24 (0.075, 0.78), *p* = 0.017	
Retired	1.01 (0.52, 1.98), *p* = 0.97		1.64 (0.65, 4.15), *p* = 0.30		0.56 (0.20, 1.59), *p* = 0.27	
Student	0.77 (0.15, 3.90), *p* = 0.75		0.63 (0.11, 3.59), *p* = 0.60		1.87 (0.00, $6.30\times 10^{5}$), *p* = 0.99	
Q10: What is the highest level of education you completed?						
Associate/Bachelor’s Degree	Referent	Referent	Referent		Referent	
Graduate Degree	2.26 (0.71, 7.14), *p* = 0.17	5.98 (0.70, 51.20), *p* = 0.10	5.02 (0.62, 40.93), *p* = 0.13		1.11 (0.23, 5.36), *p* = 0.90	
High School Degree	0.35 (0.17, 0.73), *p* = 0.005	0.79 (0.20, 3.12), *p* = 0.73	0.40 (0.15, 1.06), *p* = 0.066		0.25 (0.073, 0.86), *p* = 0.028	
Some College	0.66 (0.31, 1.42), *p* = 0.29	2.10 (0.51, 8.73), *p* = 0.31	0.67 (0.26, 1.69), *p* = 0.39		0.59 (0.15, 2.29), *p* = 0.45	
Some High School	0.65 (0.16, 2.56), *p* = 0.53	0.024 (0.0017, 0.35), *p* = 0.006	10.40 (0.00, $2.45\;x\;10^{5}$), *p* = 0.99		0.15 (0.024, 0.91), *p* = 0.040	
Trade School	0.65 (0.12, 3.33), *p* = 0.60	2.43 (0.20, 28.85), *p* = 0.48	0.96 (0.10, 9.08), *p* = 0.98		0.33 (0.028, 4.01), *p* = 0.39	
Q11: What is your marital status?						
Married	Referent		Referent		Referent	
Divorced	0.82 (0.37, 1.81), *p* = 0.62		0.59 (0.20, 1.72), *p* = 0.34		1.13 (0.34, 3.76), *p* = 0.84	
Single	0.73 (0.39, 1.38), *p* = 0.33		0.87 (0.37, 2.05), *p* = 0.75		0.59 (0.23, 1.51), *p* = 0.27	
Widowed	1.47 (0.48, 4.48), *p* = 0.50		1.63 (0.44, 6.04), *p* = 0.47		1.18 (0.13, 10.56), *p* = 0.88	
Q12: How would you describe your political view?						
Independent	Referent	Referent	Referent	Referent	Referent	Referent
Conservative	0.83 (0.41, 1.68), *p* = 0.60	0.49 (0.13, 1.85), *p* = 0.29	0.80 (0.31, 2.04), *p* = 0.64	1.07 (0.26, 4.34), *p* = 0.92	0.84 (0.29, 2.47), *p* = 0.75	0.00 (0.00, 0.50), *p* = 0.034
Liberal	2.05 (0.93, 4.54), *p* = 0.077	0.66 (0.18, 2.38), *p* = 0.52	3.79 (1.17, 12.28), *p* = 0.027	3.46 (0.77, 15.47), *p* = 0.10	1.07 (0.35, 3.26), *p* = 0.91	0.40 (0.029, 5.53), *p* = 0.090
Body Mass Index	0.98 (0.94, 1.01), *p* = 0.21	0.97 (0.91, 1.04), *p* = 0.42	1.00 (0.95, 1.05), *p* = 0.97		0.94 (0.89, 0.999), *p* = 0.041	1.46 (0.97, 2.21), *p* = 0.070
Dyslipidemia						
No Dyslipidemia	Referent		Referent		Referent	
Dyslipidemia	2.02 (1.14, 3.58), *p* = 0.016		2.18 (0.96, 4.99), *p* = 0.064		1.91 (0.84, 4.37), *p* = 0.12	
Type 1 or 2 Diabetes Mellitus						
No Diabetes Mellitus	Referent		Referent		Referent	
Diabetes Mellitus	1.17 (0.54, 2.55), *p* = 0.69		1.92 (0.54, 6.83), *p* = 0.31		0.78 (0.28, 2.16), *p* = 0.64	
Hypertension						
No Hypertension	Referent		Referent		Referent	
Hypertension	1.60 (0.91, 2.82), *p* = 0.10		1.94 (0.87, 4.34), *p* = 0.11		1.29 (0.57, 2.94), *p* = 0.54	
Coronary Artery Disease or Prior Myocardial Infarction (CAD/MI)						
No CAD/MI	Referent		Referent		Referent	
CAD/MI	0.70 (0.30, 1.65), *p* = 0.42		0.88 (0.23, 3.35), *p* = 0.85		0.58 (0.19, 1.77), *p* = 0.34	
Peripheral Vascular Disease (PVD)						
No PVD	Referent		Referent		Referent	
PVD	0.77 (0.21, 2.89), *p* = 0.70		12.66 (0.00, $1.52\times 10^{6}$), *p* = 0.99		0.28 (0.058, 1.31), *p* = 0.11	
Smoking						
Never	Referent	Referent	Referent	Referent	Referent	
Current	0.51 (0.23, 1.16), *p* = 0.11	0.22 (0.049, 0.95), *p* = 0.042	0.55 (0.19, 1.58), *p* = 0.27	0.24 (0.045, 1.29), *p* = 0.097	0.46 (0.13, 1.65), *p* = 0.23	
Former	1.14 (0.52, 2.50), *p* = 0.74	2.51 (0.44, 14.38), *p* = 0.30	5.09 (0.65, 39.57), *p* = 0.12	8.35 (0.28, 251.03), *p* = 0.22	0.60 (0.23, 1.55), *p* = 0.29	
Congestive Heart Failure (CHF)						
No CHF	Referent		Referent		Referent	
CHF	1.42 (0.17, 12.01), *p* = 0.75		9.00 (0.00, $1.51\times 10^{8}$), *p* = 0.99		0.67 (0.067, 6.69), *p* = 0.73	
History of Atrial Fibrillation or Flutter (Afib)						
No Afib	Referent		Referent		Referent	
Afib	1.69 (0.49, 5.86), *p* = 0.41		2.27 (0.28, 18.51), *p* = 0.45		1.38 (0.29, 6.55), *p* = 0.69	
Cerebrovascular Accident						
No	Referent	Referent	Referent	Referent	Referent	
Yes	2.33 (0.89, 6.13), *p* = 0.087	24.75 (1.84, 333.64), *p* = 0.016	6.46 (0.84, 49.58), *p* = 0.073	11.10 (0.001, $1.36\times 10^{5}$), *p* = 0.99	1.28 (0.40, 4.06), *p* = 0.67	
Alcohol Use Screen (Positive AUDIT-C)						
Negative Screen	Referent		Referent		Referent	
Positive Screen	0.97 (0.42, 2.22), *p* = 0.94		0.56 (0.20, 1.57), *p* = 0.27		2.23 (0.49, 10.22), *p* = 0.30	
History of Alcohol Use Disorder (DSM Diagnosed)						
No Alcohol Use Disorder	Referent		Referent		Referent	
Alcohol Use Disorder	1.69 (0.20, 13.98), *p* = 0.63		0.037 (0.00, 2.58), *p* = 0.99		12.61 (0.00, 622,517.45), *p* = 0.99	
Depression Screen (Positive PHQ-9)						
Negative Screen	Referent		Referent		Referent	Referent
Positive Screen	0.68 (0.29, 1.60), *p* = 0.38		0.97 (0.26, 3.65), *p* = 0.96		0.49 (0.16, 1.55), *p* = 0.23	0.43 (0.047, 3.91), *p* = 0.093
History of Psychiatric Disorder (DSM Diagnosed)						
No Psychiatric History	Referent		Referent		Referent	
Psychiatric History	1.20 (0.68, 2.11), *p* = 0.54		1.16 (0.55, 2.44), *p* = 0.70		1.30 (0.53, 3.18), *p* = 0.57	
Illicit Drug Use						
No	Referent		Referent		Referent	
Yes	0.32 (0.13, 0.82), *p* = 0.018		0.39 (0.088, 1.71), *p* = 0.21		0.27 (0.078, 0.92), *p* = 0.037	
Peptic Ulcer Disease (PUD)						
No PUD	Referent		Referent		Referent	
PUD	2.32 (0.53, 10.22), *p* = 0.27		2.54 (0.31, 20.50), *p* = 0.38		2.09 (0.25, 17.24), *p* = 0.49	
Liver Disease (i.e., Cirrhosis)						
No Liver Disease	Referent		Referent		Referent	
Liver Disease	1.66 (0.20, 13.71), *p* = 0.64		9.00 (0.00, $1.51\times 10^{8}$), *p* = 0.99		0.89 (0.096, 8.31), *p* = 0.92	
Connective Tissue Disease (CTD)						
No CTD	Referent		Referent		Referent	
CTD	11.18 (0.00, $4.04\times 10^{6}$), *p* = 0.98		9.00 (0.00, $1.51\times 10^{8}$), *p* = 0.99		3.62 (0.00, $7.08\times 10^{8})$, *p* = 0.99	
Chronic Pulmonary Disease						
No Chronic Pulmonary Disease	Referent		Referent		Referent	
Chronic Pulmonary Disease	1.36 (0.58, 3.19), *p* = 0.49		1.14 (0.40, 3.25), *p* = 0.81		1.91 (0.41, 8.82), *p* = 0.41	
Hemiplegia						
No Hemiplegia	Referent		Referent		Referent	
Hemiplegia	0.93 (0.19, 4.51), *p* = 0.93		0.48 (0.042, 5.44), *p* = 0.55		1.36 (0.16, 11.78), *p* = 0.78	
Dementia						
No Dementia	Referent		Referent		Referent	
Dementia	1.80 (0.40, 8.09), *p* = 0.44		10.85 (0.00, $1.90\times 10^{7}$), *p* = 0.99		1.25 (0.26, 6.00), *p* = 0.78	
Moderate to Severe Renal Disease						
No Renal Disease	Referent		Referent		Referent	
Renal Disease	4.98 (0.66, 37.82), *p* = 0.12		14.92 (0.01, 40,598.69), *p* = 0.99		1.60 (0.19, 13.57), *p* = 0.67	
Solid Tumor (Localized or Metastatic)						
No Tumor	Referent		Referent		Referent	
Tumor	0.67 (0.31, 1.44), *p* = 0.30		1.47 (0.47, 4.57), *p* = 0.50		0.22 (0.067, 0.71), *p* = 0.011	
Autoimmune Disease						
No Autoimmune Disease	Referent		Referent		Referent	
Autoimmune Disease	1.52 (0.44, 5.30), *p* = 0.51		1.78 (0.38, 8.21), *p* = 0.46		1.13 (0.13, 10.02), *p* = 0.92	
Thyroid Disease						
No Thyroid Disease	Referent		Referent		Referent	
Thyroid Disease	1.33 (0.53, 3.32), *p* = 0.54		1.02 (0.38, 2.70), *p* = 0.98		12.71 (0.001, 307,943.72), *p* = 0.99	
Musculoskeletal Disorder (MSK)						
No MSK Disorder	Referent	Referent	Referent		Referent	
MSK Disorder	1.93 (1.11, 3.35), *p* = 0.020	2.08 (0.72, 6.00), *p* = 0.18	2.23 (1.05, 4.70), *p* = 0.036		1.61 (0.71, 3.67), *p* = 0.26	
Charlson Comorbidity Index (CCI)	1.21 (1.05, 1.39), *p* = 0.008		1.49 (1.18, 1.88), *p* = 0.001	1.28 (0.87, 1.87), *p* = 0.21	1.01 (0.84, 1.23), *p* = 0.90	
10-Year Survival CCI	0.99 (0.98, 1.00), *p* = 0.03		0.97 (0.95, 0.99), *p* = 0.004		1.00 (0.99, 1.01), *p* = 0.77	

**Table 9:** Univariate and multivariable analysis of variables associated with vaccine hesitancy for patients with neurological disorders stratified by race.

	White Patients		Asian Patients		Native Hawaiian or Other Pacific Islander	
	Unadjusted Odds Ratios (95% CI)	Best Fit Model: Adjusted Odds Ratios	Unadjusted Odds Ratios (95% CI)	Best Fit Model: Adjusted Odds Ratios	Unadjusted Odds Ratios (95% CI)	Best Fit Model: Adjusted Odds Ratios
Age	1.03 (1.00, 1.06), *p* = 0.049	1.05 (0.98, 1.13), *p* = 0.16	1.01 (0.98, 1.04), *p* = 0.43		1.02 (0.99, 1.05), *p* = 0.14	
Median Household Income	1.00 (1.00, 1.00), *p* = 0.69		1.00 (1.00, 1.00), *p* = 0.96		1.00 (1.00, 1.00), *p* = 0.49	
Overall Poverty Level	$38.12\;(0.00,\;1.11\times 10^{7}$), *p* = 0.57		0.40 (0.00, 361,835.57), *p* = 0.89		0.002 (0.00, 178.37), *p* = 0.28	
Poverty Level Ages 18–64	53.11 (0.00, $3.08\times 10^{7}$), *p* = 0.56		7.23 (0.00, $2.62\times 10^{7}$), *p* = 0.80		0.0014 (0.00, 313.60), *p* = 0.30	
Poverty Level 65 and Older	0.84 (0.00, 25,882.48), *p* = 0.97		0.0019 (0.00, 99.69), *p* = 0.26		0.00 (0.00, 11.22), *p* = 0.12	0.00 (0.00, 1003.37), *p* = 0.13
Origin Population Size	1.00 (1.00, 1.00), *p* = 0.67		1.00 (1.00, 1.00), *p* = 0.36		1.00 (1.00, 1.00), *p* = 0.20	
Geographic Origin						
Urban	Referent		Referent		Referent	
Suburban	0.86 (0.34, 2.22), *p* = 0.76		1.32 (0.45, 3.88), *p* = 0.62		1.50 (0.53, 4.23), *p* = 0.44	
Rural	0.45 (0.043, 4.81), *p* = 0.51		NA		1.16 (0.00, 55,495.19), *p* = 0.99	
Income Quartiles						
Third Quartile (Middle Class)	Referent		Referent		Referent	
First Quartile	0.92 (0.19, 4.39), *p* = 0.92		0.43 (0.07, 2.63), *p* = 0.36		0.48 (0.079, 2.95), *p* = 0.43	
Second Quartile	0.36 (0.091, 1.38), *p* = 0.14		0.27 (0.05, 1.52), *p* = 0.14		0.81 (0.14, 4.82), *p* = 0.82	
Fourth Quartile	0.29 (0.08, 1.05), *p* = 0.06		0.29 (0.05, 1.60), *p* = 0.15		0.48 (0.079, 2.95), *p* = 0.43	
Insurance						
Private	Referent		Referent		Referent	
Medicaid	0.51 (0.16, 1.68), *p* = 0.27		1.20 (0.28, 5.10), *p* = 0.80		0.43 (0.13, 1.43), *p* = 0.17	
Medicare	1.80 (0.56, 5.80), *p* = 0.33		1.67 (0.46, 6.03), *p* = 0.43		0.78 (0.19, 3.26), *p* = 0.73	
Military	4.91 (0.59, 41.08), *p* = 0.14		0.071 (0.003, 2.01), *p* = 0.99		0.72 (0.063, 8.20), *p* = 0.79	
Sex						
Female	Referent		Referent	Referent	Referent	
Male	1.22 (0.47, 3.16), *p* = 0.68		2.54 (0.83, 7.78), *p* = 0.10	2.79 (0.60, 12.97), *p* = 0.19	0.62 (0.22, 1.75), *p* = 0.37	
Q1: Have you had a one-on-one discussion with a physician about the risks and benefits of receiving the COVID vaccination?						
Had Conversation	Referent		Referent		Referent	
No Conversation	0.54 (0.15, 1.98), *p* = 0.36		0.68 (0.18, 2.61), *p* = 0.57		0.47 (0.096, 2.36), *p* = 0.36	
Q2: Primary source of COVID information						
Scholarly Articles/ CDC/ US Governmental Agencies	Referent		Referent		Referent	
Healthcare Provider	0.58 (0.05, 6.57), *p* = 0.66		1.50 (0.09, 25.39), *p* = 0.78		9.62 (0.00, $1.74\times 10^{7}$), *p* = 0.99	
Friends/Family/Coworkers	0.42 (0.075, 2.36), *p* = 0.32		2.25 (0.23, 22.14), *p* = 0.49		0.30 (0.048, 1.88), *p* = 0.20	
Traditional Media (TV News, Radio, Print Media)	0.69 (0.18, 2.67), *p* = 0.59		3.31 (0.52, 21.13), *p* = 0.21		0.83 (0.15, 4.53), *p* = 0.82	
Social Media	0.16 (0.025, 1.03), *p* = 0.054		1.13 (0.14, 8.88), *p* = 0.91		0.13 (0.013, 1.39), *p* = 0.09	
Q3: Do you believe that vaccines are safe?						
Yes	Referent	Referent	Referent	Referent	Referent	Referent
No	0.094 (0.027, 0.32), *p* < 0.001	0.039 (0.002, 0.91), *p* = 0.04	0.064 (0.011, 0.37), *p* = 0.002	0.065 (0.009, 0.47), *p* = 0.007	0.11 (0.030, 0.39), *p* = 0.001	0.0004 (0.00, 1.04), *p* = 0.051
Q4: Do you believe that COVID is a severe illness?						
Yes	Referent	Referent	Referent		Referent	
No	0.081 (0.021, 0.32), *p* < 0.001	0.032 (0.00, 2.51), *p* = 0.12	1.58 (0.18, 13.74), *p* = 0.68		0.22 (0.060, 0.84), *p* = 0.027	
Q5: Do you have a preexisting medical condition that you believe will make the vaccine unsafe?						
Yes	Referent	Referent	Referent	Referent	Referent	
No	9.45 (3.30, 27.04), *p* < 0.001	11.37 (1.01, 127.98), *p* = 0.049	8.13 (2.59, 25.49), *p* < 0.001	5.53 (1.41, 21.67), *p* = 0.014	1.94 (0.65, 5.75), *p* = 0.23	
Q6: Have you received the flu vaccine within the last year?						
Yes	Referent	Referent	Referent		Referent	
No	0.12 (0.042, 0.36), *p* < 0.001	0.12 (0.01, 1.58), *p* = 0.11	0.20 (0.065, 0.59), *p* = 0.004		0.41 (0.15, 1.18), *p* = 0.098	
Q7: Have you tested positive for COVID?						
Yes	Referent		Referent		Referent	
No	39.44 (0.51, 3050.53), *p* = 0.99		4.53 (0.27, 76.08), *p* = 0.29		0.28 (0.00, 13,449.63), *p* = 0.99	
Q9: How would you define your work status?						
Employed	Referent		Referent		Referent	
Unemployed	1.05 (0.11, 10.10), *p* = 0.97		7.56 (0.00, $1.11\times 10^{8}$), *p* = 0.99		0.80 (0.070, 9.18), *p* = 0.86	
Homemaker	0.75 (0.13, 4.29), *p* = 0.75		0.21 (0.012, 3.93), *p* = 0.30		0.32 (0.00, 2629.21), *p* = 0.99	
Not Able to Work	0.48 (0.13, 1.80), *p* = 0.28		0.29 (0.050, 1.62), *p* = 0.16		0.40 (0.10, 1.57), *p* = 0.19	
Retired	1.16 (0.36, 3.71), *p* = 0.81		1.16 (0.35, 3.84), *p* = 0.80		0.50 (0.12, 2.09), *p* = 0.34	
Student	0.27 (0.00, 862.57), *p* = 0.99		0.64 (0.057, 7.29), *p* = 0.72		0.40 (0.029, 5.55), *p* = 0.49	
Q10: What is the highest level of education you completed?						
Associate/Bachelor’s Degree	Referent	Referent	Referent		Referent	
Graduate Degree	3.89 (0.43, 34.82), *p* = 0.22	11.70 (0.01, 11181.86), *p* = 0.99	1.54 (0.27, 8.64), *p* = 0.62		0.64 (0.00, 5592.09), *p* = 0.99	
High School Degree	0.15 (0.042, 0.56), *p* = 0.005	0.036 (0.0015, 0.82), *p* = 0.04	0.90 (0.24, 3.31), *p* = 0.87		0.33 (0.061, 1.81), *p* = 0.20	
Some College	0.64 (0.17, 2.42), *p* = 0.52	0.64 (0.033, 12.27), *p* = 0.77	2.80 (0.52, 14.99), *p* = 0.23		0.15 (0.027, 0.85), *p* = 0.032	
Some High School	0.33 (0.029, 3.84), *p* = 0.38	0.027 (0.00, 6.18), *p* = 0.19	12.81 (0.00, $2.34\times 10^{7}$), *p* = 0.99		0.44 (0.032, 6.19), *p* = 0.55	
Trade School	2.29 (0.001, 4435.52), *p* = 0.99	10.70 (0.0033, 34834.71), *p* = 1.00	0.56 (0.044, 7.12), *p* = 0.65		0.11 (0.005, 2.55), *p* = 0.17	
Q11: What is your marital status?						
Married	Referent		Referent		Referent	
Divorced	0.58 (0.16, 2.08), *p* = 0.40		0.47 (0.099, 2.19), *p* = 0.33		1.25 (0.28, 5.60), *p* = 0.77	
Single	0.43 (0.15, 1.29), *p* = 0.13		1.40 (0.34, 5.67), *p* = 0.64		1.02 (0.32, 3.24), *p* = 0.98	
Widowed	1.09 (0.12, 9.67), *p* = 0.94		1.40 (0.27, 7.25), *p* = 0.69		1.88 (0.19, 18.32), *p* = 0.59	
Q12: How would you describe your political view?						
Independent	Referent	Referent	Referent		Referent	Referent
Conservative	0.89 (0.28, 2.82), *p* = 0.84	0.074 (0.003, 1.58), *p* = 0.096	0.61 (0.15, 2.46), *p* = 0.49		0.47 (0.10, 2.15), *p* = 0.33	0.047 (0.001, 2.75), *p* = 0.14
Liberal	2.61 (0.65, 10.47), *p* = 0.18	0.21 (0.01, 3.61), *p* = 0.28	3.33 (0.34, 32.27), *p* = 0.30		2.38 (0.56, 10.01), *p* = 0.24	0.01 (0.00, 5.12), *p* = 0.15
Body Mass Index	1.06 (0.97, 1.15), *p* = 0.18		0.92 (0.84, 1.01), *p* = 0.078	0.88 (0.78, 0.99), *p* = 0.032	0.96 (0.89, 1.02), *p* = 0.19	0.92 (0.73, 1.17), *p* = 0.51
Dyslipidemia						
No Dyslipidemia	Referent		Referent		Referent	Referent
Dyslipidemia	3.78 (1.20, 11.90), *p* = 0.023		1.40 (0.50, 3.94), *p* = 0.52		2.22 (0.71, 6.87), *p* = 0.17	28.54 (0.51, 1605.85), *p* = 0.10
Type 1 or 2 Diabetes Mellitus						
No Diabetes Mellitus	Referent		Referent		Referent	
Diabetes Mellitus	0.52 (0.15, 1.79), *p* = 0.30		5.25 (0.65, 42.43), *p* = 0.12		1.52 (0.38, 6.12), *p* = 0.56	
Hypertension						
No Hypertension	Referent		Referent		Referent	
Hypertension	3.92 (1.09, 14.03), *p* = 0.036		1.25 (0.44, 3.52), *p* = 0.67		1.25 (0.42, 3.76), *p* = 0.69	
Coronary Artery Disease or Prior Myocardial Infarction (CAD/MI)						
No CAD/MI	Referent		Referent		Referent	
CAD/MI	0.50 (0.10, 2.69), *p* = 0.42		1.44 (0.29, 7.17), *p* = 0.65		0.49 (0.13, 1.95), *p* = 0.32	
Peripheral Vascular Disease (PVD)						
No PVD	Referent		Referent		Referent	
PVD	1.23 (0.00, 202,279.46), *p* = 0.99		0.49 (0.042, 5.68), *p* = 0.57		0.96 (0.17, 5.25), *p* = 0.96	
Smoking						
Never	Referent	Referent	Referent		Referent	
Current	0.24 (0.07, 0.84), *p* = 0.025	0.06 (0.003, 1.19), *p* = 0.065	1.02 (0.11, 9.84), *p* = 0.99		0.65 (0.16, 2.57), *p* = 0.54	
Former	0.63 (0.18, 2.18), *p* = 0.47	1.05 (0.046, 24.00), *p* = 0.97	1.02 (0.25, 4.11), *p* = 0.98		1.78 (0.34, 9.29), *p* = 0.49	
Congestive Heart Failure (CHF)						
No CHF	Referent		Referent		Referent	
CHF	1.23 (0.00, 202,279.46), *p* = 0.99		0.24 (0.01, 4.08), *p* = 0.33		13.57 (0.00, $2.32\times 10^{8}$), *p* = 0.99	
History of Atrial Fibrillation or Flutter (Afib)						
No Afib	Referent		Referent		Referent	
Afib	10.41 (0.001, 133927.77), *p* = 0.99		1.00 (0.10, 9.53), *p* = 1.00		1.14 (0.21, 6.10), *p* = 0.88	
Cerebrovascular Accident						
No	Referent		Referent		Referent	
Yes	12.50 (0.02, 8543.15), *p* = 0.99		0.81 (0.20, 3.30), *p* = 0.77		2.69 (0.55, 13.29), *p* = 0.22	
Alcohol Use Screen (Positive AUDIT-C)						
Negative Screen	Referent		Referent		Referent	
Positive Screen	0.63 (0.20, 1.92), *p* = 0.41		0.73 (0.13, 3.95), *p* = 0.71		15.42 (0.00, $2.55\times 10^{7}$), *p* = 0.99	
History of Alcohol Use Disorder (DSM Diagnosed)						
No Alcohol Use Disorder	Referent		Referent		Referent	
Alcohol Use Disorder	0.90 (0.10, 8.12), *p* = 0.93		NA		9.66 (0.00, $8.59\times 10^{9}$), *p* = 0.99	
Depression Screen (Positive PHQ-9)						
Negative Screen	Referent		Referent		Referent	Referent
Positive Screen	1.94 (0.23, 16.04), *p* = 0.54		1.13 (0.22, 5.76), *p* = 0.89		0.12 (0.024, 0.58), *p* = 0.009	0.003 (0.00, 0.78), *p* = 0.040
History of Psychiatric Disorder (DSM Diagnosed)						
No Psychiatric History	Referent		Referent		Referent	
Psychiatric History	1.84 (0.69, 4.88), *p* = 0.22		1.14 (0.36, 3.60), *p* = 0.82		0.63 (0.21, 1.90), *p* = 0.41	
Illicit Drug Use						
No	Referent	Referent	Referent		Referent	Referent
Yes	0.19 (0.06, 0.61), *p* = 0.005	13.98 (0.39, 507.02), *p* = 0.15	5.75 (0.00, $3.14\times 10^{9})$, *p* = 0.99		0.30 (0.039, 2.31), *p* = 0.25	0.069 (0.00$,\;7.92\times 10^{6}$), *p* = 0.78
Peptic Ulcer Disease (PUD)						
No PUD	Referent		Referent		Referent	
PUD	1.64 (0.20, 13.63), *p* = 0.65		1.83 (0.21, 15.91), *p* = 0.58		13.57 (0.00, $2.2\times 10^{8}$), *p* = 0.99	
Liver Disease (i.e., Cirrhosis)						
No Liver Disease	Referent		Referent		Referent	
Liver Disease	0.34 (0.030, 3.95), *p* = 0.39		9.76 (0.00, $1.67\times 10^{8}$), *p* = 0.99		9.66 (0.00, $8.59\times 10^{9}$), *p* = 0.99	
Connective Tissue Disease (CTD)						
No CTD	Referent		Referent		Referent	
CTD	1.23 (0.00, 202,279.46), *p* = 0.99		0.37 (0.00, 3684.10), *p* = 0.99		9.66 (0.00, $8.59\times 10^{9}$), *p* = 0.99	
Chronic Pulmonary Disease						
No Chronic Pulmonary Disease	Referent		Referent		Referent	
Chronic Pulmonary Disease	1.14 (0.31, 4.25), *p* = 0.85		1.83 (0.21, 15.91), *p* = 0.58		1.53 (0.30, 7.91), *p* = 0.61	
Hemiplegia						
No Hemiplegia	Referent		Referent		Referent	
Hemiplegia	0.52 (0.051, 5.22), *p* = 0.58		0.37 (0.00, 3684.10), *p* = 0.99		1.31 (0.14, 12.55), *p* = 0.82	
Dementia						
No Dementia	Referent		Referent		Referent	
Dementia	1.25 (0.15, 10.72), *p* = 0.84		1.55 (0.17, 13.71), *p* = 0.70		9.66 (0.00, $8.59\times 10^{9}$), *p* = 0.99	
Moderate to Severe Renal Disease						
No Renal Disease	Referent		Referent		Referent	
Renal Disease	10.41 (0.001, 133,927.77), *p* = 0.99		12.88 (0.00, $4.88\times 10^{6}$), *p* = 0.99		2.04 (0.23, 18.28), *p* = 0.52	
Solid Tumor (Localized or Metastatic)						
No Tumor	Referent		Referent		Referent	Referent
Tumor	0.93 (0.25, 3.53), *p* = 0.92		2.13 (0.25, 18.18), *p* = 0.49		0.14 (0.030, 0.69), *p* = 0.015	0.00 (0.00, 2.29), *p* = 0.079
Autoimmune Disease						
No Autoimmune Disease	Referent		Referent		Referent	
Autoimmune Disease	0.78 (0.16, 3.88), *p* = 0.76		12.88 (0.00, $4.88\times 10^{6}$), *p* = 0.99		0.96 (0.093, 9.89), *p* = 0.97	
Thyroid Disease						
No Thyroid Disease	Referent		Referent		Referent	
Thyroid Disease	1.36 (0.37, 5.03), *p* = 0.64		0.45 (0.10, 2.03), *p* = 0.30		15.42 (0.00, $2.55\times 10^{7}$), *p* = 0.99	
Musculoskeletal Disorder (MSK)						
No MSK Disorder	Referent	Referent	Referent		Referent	
MSK Disorder	3.85 (1.44, 10.29), *p* = 0.007	8.44 (0.78, 91.37), *p* = 0.079	1.03 (0.37, 2.88), *p* = 0.96		1.30 (0.45, 3.81), *p* = 0.63	
Charlson Comorbidity Index (CCI)	1.25 (0.97, 1.60), *p* = 0.082		1.16 (0.90, 1.50), *p* = 0.26		1.11 (0.86, 1.45), *p* = 0.42	
10-Year Survival CCI	0.99 (0.97, 1.01), *p* = 0.18		0.99 (0.97, 1.01), *p* = 0.29		0.99 (0.98, 1.01), *p* = 0.57	

**Table 10:** Unadjusted odds of vaccine acceptance or hesitance amongst neurological patients.

Vaccination Odds Reduced	Entire Cohort	Female	Male	White	Asian	NHPI
Believes Vaccine Not Safe	✓	✓	✓	✓	✓	✓
Self-Perceives Contraindicated Preexisting Condition	✓	✓	✓	✓	✓	
Did Not Receive Annual Influenza Vaccine	✓	✓	✓	✓	✓	
Believes COVID-19 Not Severe	✓	✓	✓	✓		
Younger Age	✓	✓		✓		
Higher CCI	✓	✓		✓		
Illicit Drug Use	✓			✓		
Solid Tumors			✓			✓
Depression Screen Positive						✓
Medicaid Insurance	✓		✓			
Military Insurance					✓	
Not Able to Work			✓			
Social Media	✓		✓			
High School Degree		✓	✓	✓		
NHPI			✓			
**Vaccination Odds Increased**	**Entire Cohort**	**Female**	**Male**	**White**	**Asian**	**NHPI**
Politically Liberal	✓	✓				
Traditional Media	✓		✓			
Graduate Degree		✓		✓		
3rd Income Quartile	✓					
Medicare Insurance		✓				
Musculoskeletal Disorder	✓			✓		
Dyslipidemia	✓			✓		
Hypertension				✓		

**Table 11:** Strongest predictors of vaccination acceptance or hesitance amongst neurologic patients, according to multivariable logistic regression.

Vaccination Odds Reduced	Entire Cohort	Female	Male	White	Asian	NHPI
Believes Vaccine Not Safe	✓	✓		✓	✓	
Self-Perceives Contraindicated Preexisting Condition	✓	✓		✓	✓	
Did Not Receive Annual Influenza Vaccine	✓	✓	✓			
Believes COVID-19 Not Severe			✓			
Politically Conservative			✓			
Social Media	✓					
High School Degree				✓		
Some High School	✓					
Current Smoker	✓					
Higher Body Mass Index					✓	
Depression Screen Positive						✓
**Vaccination Odds Increased**	**Entire Cohort**	**Female**	**Male**	**White**	**Asian**	**NHPI**
Cerebrovascular Accident	✓					

## Data Availability

The raw data supporting the conclusions of this article will be made available by the authors, without undue reservation.

## Supplementary Materials

The following are available online at https://www.mdpi.com/article/10.3390/idr13030072/s1, Table S1: Comparison of patients providing complete or partial surveys (participants) to those who did not participate (i.e., non-response, declined, or break-offs). Relative to non-participants, Whites and married patients had a greater odds of survey participation; NAANs were at reduced odds of survey participation.

## Appendix A

Thirteen-Question Quality Improvement Survey

1. Do you plan on getting the COVID-19 vaccine?
  (a) COVID vaccine received
  (b) Planning to receive as soon as available
  (c) Planning to receive within the year
  (d) Not planning to receive
  (e) Decline to answer
2. Have you had a one-on-one discussion with a physician about the risks and benefits of receiving the COVID vaccination?
  (a) Yes
  (b) No
  (c) Decline to answer
3. What is your primary source of COVID information? Please listen to all of the options before answering.
  (a) Social media
  (b) Media news (TV, radio, articles)
  (c) Friends/family/coworkers
  (d) Healthcare provider
  (e) Scholarly articles (including websites from CDC or other government agencies)
  (f) Decline to answer
4. Do you believe that vaccines are safe?
  (a) Yes
  (b) No
  (c) Decline to answer
5. Do you believe that COVID is a severe illness?
  (a) Yes
  (b) No
  (c) Decline to answer
6. Do you have a pre-existing medical condition that you believe will make the vaccine unsafe?
  (a) Yes
  (b) No
  (c) Decline to answer
7. Have you received the flu vaccine within the last year?
  (a) Yes
  (b) No
  (c) Decline to answer
8. Have you tested-positive for COVID?
  (a) Yes
  (b) No
  (c) Decline to answer
9. With a single category, how would you define your race/ethnicity?
  (a) White
  (b) Black
  (c) Asian
  (d) Hispanic
  (e) Native Hawaiian or Other Pacific Islander
  (f) Native American or Alaskan Native
  (g) Decline to answer
10. How would you define your work status?
  (a) Employed Full-Time
  (b) Employed Part-Time
  (c) Seeking Job Opportunities
  (d) Homemaker
  (e) Student
  (f) Military/Forces
  (g) Retired
  (h) Not able to work
  (i) Decline to answer
11. What is the highest level of education you completed?
  (a) Some high school
  (b) High school or GED
  (c) Trade School
  (d) Some college
  (e) Associate or bachelor’s degree
  (f) Master’s degree
  (g) Doctorate degree
  (h) Decline to answer
12. What is your marital status?
  (a) Single
  (b) Married
  (c) Divorced
  (d) Widowed
  (e) Decline to answer
13. How would you describe your political view?
  (a) Very Liberal
  (b) Moderately Liberal
  (c) Slightly Liberal
  (d) Slightly Conservative
  (e) Moderately Conservative
  (f) Very Conservative
  (g) Decline to answer

## References

[1] Szilagyi P.G., Thomas K., Shah M.D., Vizueta N., Cui Y., Vangala S., Kapteyn A. (2021). National Trends in the US Public’s Likelihood of Getting a COVID-19 Vaccine—1 April to 8 December, 2020. JAMA.

[2] MacDonald N.E. (2015). Vaccine hesitancy: Definition, scope and determinants. Vaccine.

[3] Mello M.M., Silverman R.D., Omer S.B. (2020). Ensuring Uptake of Vaccines against SARS-CoV-2. N. Engl. J. Med..

[4] Hollmeyer H., Hayden F., Mounts A., Buchholz U. (2013). Review: Interventions to increase influenza vaccination among healthcare workers in hospitals. Influenza Other Respir. Viruses.

[5] Ro J.M., Yee A.K. (2010). Out of the shadows: Asian Americans, Native Hawaiians, and Pacific Islanders. Am. J. Public Health.

[6] Williamson E.J., Walker A.J., Bhaskaran K., Bacon S., Bates C., Morton C.E., Curtis H.J., Mehrkar A., Evans D., Inglesby P. (2020). Factors associated with COVID-19-related death using OpenSAFELY. Nature.

[7] Rosenthal N., Cao Z., Gundrum J., Sianis J., Safo S. (2020). Risk Factors Associated With In-Hospital Mortality in a US National Sample of Patients With COVID-19. JAMA Netw. Open.

[8] GBD 2016 Neurology Collaborators (2019). Global, regional, and national burden of neurological disorders, 1990–2016: A systematic analysis for the Global Burden of Disease Study 2016. Lancet Neurol..

[9] Ruiz B.J., Bell R.A. (2021). Predictors of intention to vaccinate against COVID-19: Results of a nationwide survey. Vaccine.

[10] Kreps S., Prasad S., Brownstein J.S., Hswen Y., Garibaldi B.T., Zhang B., Kriner D.L. (2020). Factors Associated with US Adults’ Likelihood of Accepting COVID-19 Vaccination. JAMA Netw. Open.

[11] Schwarzinger M., Watson V., Arwidson P., Alla P.F., Luchini S. (2021). COVID-19 vaccine hesitancy in a representative working-age population in France: A survey experiment based on vaccine characteristics. Lancet Public Health.

[12] Lazarus J.V., Ratzan S.C., Palayew A., Gostin L.O., Larson H.J., Rabin K., Kimball S., El-Mohandes A. (2021). A global survey of potential acceptance of a COVID-19 vaccine. Nat. Med..

[13] Frankel L.R. (1983). The report of the CASRO task force on response rates. Improving Data Quality in a Sample Survey.

[14] Smith M., Nakamoto M., Crocker J., Morden F.T., Liu K., Ma E., Chong A., Van N., Vajjala V., Carrazana E. (2021). Early impact of the COVID-19 pandemic on outpatient migraine care in Hawaii: Results of a quality improvement survey. Headache J. Head Face Pain.

[15] Ghaffari-Rafi A., Gorenflo R., Hu H., Viereck J., Liow K. (2020). Role of psychiatric, cardiovascular, socioeconomic, and demographic risk factors on idiopathic normal pressure hydrocephalus: A retrospective case-control study. Clin. Neurol. Neurosurg..

[16] Lenth R.V. (2001). Some Practical Guidelines for Effective Sample Size Determination. Am. Stat..

[17] Agency for Healthcare Research and Quality Overview of the Nationwide Inpatient Sample (NIS): Healthcare and Utilization Project. https://www.hcup-us.ahrq.gov/nisoverview.jsp.

[18] Steiner C., Elixhauser A., Schnaier J. (2002). The healthcare cost and utilization project: An overview. Eff. Clin. Pract..

[19] Association A.P. (2013). Diagnostic and Statistical Manual of Mental Disorders (DSM-5®).

[20] Kroenke K., Spitzer R.L., Williams J.B. (2003). The Patient Health Questionnaire-2: Validity of a two-item depression screener. Med. Care.

[21] Babor T.F., Higgins-Biddle J.C., Saunders J.B., Monteiro M.G. (2001). The Alcohol Use Disorders Identification Test: Guidelines for Use in Primary Care.

[22] NICE (2010). Alcohol-Use Disorders: Preventing the Development of Hazardous and Harmful Drinking.

[23] Bush K., Kivlahan D.R., McDonell M.B., Fihn S.D., Bradley K.A., ACQUIP (1998). The AUDIT alcohol consumption questions (AUDIT-C): An effective brief screening test for problem drinking. Ambulatory Care Quality Improvement Project (ACQUIP). Alcohol Use Disorders Identification Test. Arch. Intern. Med..

[24] Reinert F.D., Allen J.P. (2007). The alcohol use disorders identification test: An update of research findings. Alcohol Clin. Exp. Res..

[25] Kriston L., Hölzel L., Weiser A.-K., Berner M.M., Härter M. (2008). Meta-analysis: Are 3 questions enough to detect unhealthy alcohol use?. Ann. Intern. Med..

[26] Bradley K.A., DeBenedetti A.F., Volk R.J., Williams E.C., Frank D., Kivlahan D.R. (2007). AUDIT-C as a brief screen for alcohol misuse in primary care. Alcohol. Clin. Exp. Res..

[27] Frank D., DeBenedetti A.F., Volk R.J., Williams E.C., Kivlahan D.R., Bradley K.A. (2008). Effectiveness of the AUDIT-C as a screening test for alcohol misuse in three race/ethnic groups. J. Gen. Intern. Med..

[28] Charlson M.E., Pompei P., Ales K.L., MacKenzie C.R. (1987). A new method of classifying prognostic comorbidity in longitudinal studies: Development and validation. J. Chronic Dis..

[29] Quan H., Li B., Couris C.M., Fushimi K., Graham P., Hider P., Januel J.-M., Sundararajan V. (2011). Updating and validating the Charlson comorbidity index and score for risk adjustment in hospital discharge abstracts using data from 6 countries. Am. J. Epidemiol..

[30] Radovanovic D., Seifert B., Urban P., Eberli F.R., Rickli H., Bertel O., Puhan M.A., Erne P. (2014). Validity of Charlson Comorbidity Index in patients hospitalised with acute coronary syndrome. Insights from the nationwide AMIS Plus registry 2002–2012. Heart.

[31] Wilcoxon F. (1945). Individual Comparisons by Ranking Methods. Biom. Bull..

[32] McHugh M.L. (2013). The chi-square test of independence. Biochem. Med..

[33] McDonald J. (2009). Handbook of Biological Statistics: Fisher’s Exact Test of Independence.

[34] Haldane J.B. (1956). The estimation and significance of the logarithm of a ratio of frequencies. Ann. Hum. Genet..

[35] Anscombe F.J. (1956). On estimating binomial response relations. Biometrika.

[36] Firth D. (1993). Bias reduction of maximum likelihood estimates. Biometrika.

[37] Akaike H. (1987). Factor analysis and AIC. Psychometrika.

[38] Akaike H. (1979). A Bayesian extension of the minimum AIC procedure of autoregressive model fitting. Biometrika.

[39] Akaike H. (1974). A new look at the statistical model identification. IEEE Trans. Autom. Control.

[40] McFadden D. (1973). Conditional Logit Analysis of Qualitative Choice Behavior.

[41] R Core Team (2020). R: A Language and Environment for Statistical Computing.

[42] Kaholokula J.K.A., Samoa R.A., Miyamoto R.E.S., Palafox N., Daniels S.-A. (2020). COVID-19 Special Column: COVID-19 Hits Native Hawaiian and Pacific Islander Communities the Hardest. Hawai’i J. Health Soc. Welf..

[43] Braveman P., Egerter S., Williams D.R. (2011). The social determinants of health: Coming of age. Annu. Rev. Public Health.

[44] Skjefte M., Ngirbabul M., Akeju O., Escudero D., Hernandez-Diaz S., Wyszynski D.F., Wu J.W. (2021). COVID-19 vaccine acceptance among pregnant women and mothers of young children: Results of a survey in 16 countries. Eur. J. Epidemiol..

[45] Adhikari H.E., Spong C.Y. (2021). COVID-19 Vaccination in Pregnant and Lactating Women. JAMA.

[46] Heath T.P., Le Doare K., Khalil A. (2020). Inclusion of pregnant women in COVID-19 vaccine development. Lancet Infect. Dis..

[47] Murphy J., Vallières F., Bentall R.P., Shevlin M., McBride O., Hartman T.K., McKay R., Bennett K., Mason L., Gibson-Miller J. (2021). Psychological characteristics associated with COVID-19 vaccine hesitancy and resistance in Ireland and the United Kingdom. Nat. Commun..

[48] ACOG (2021). Vaccinating Pregnant and Lactating Patients Against COVID-19.

[49] SMFM (2020). Society for Maternal-Fetal Medicine (SMFM) Statement: SARS-CoV-2 Vaccination in Pregnancy.

[50] Lu P.-J., O’Halloran A., Williams W.W. (2015). Impact of Health Insurance Status on Vaccination Coverage Among Adult Populations. Am. J. Prev. Med..

[51] Santoli J.M., Huet N.J., Smith P.J., Barker L.E., Rodewald L.E., Inkelas M., Olson L.M., Halfon N. (2004). Insurance status and vaccination coverage among US preschool children. Pediatrics.

[52] Ku L., Wachino V. (2005). The Effect of Increased Cost-Sharing in Medicaid: A Summary of Research Findings.

[53] Powell V., Saloner B., Sabik L.M. (2016). Cost Sharing in Medicaid: Assumptions, Evidence, and Future Directions. Med. Care Res. Rev..

[54] Families B.S. (2020). Military Families’ Perceptions of the COVID-19 Vaccine. Pulse Check.

[55] Social Security (2017). Research, Statistics & Policy Analysis. Middle Class Beneficiaries, 2014.

[56] Occupational Safety and Health Administration (OSHA) (2020). Worker Exposure Risk to COVID-19.

[57] Montagni I., Ouazzani-Touhami K., Mebarki A., Texier N., Schück S., Tzourio C., the CONFINS group (2021). Acceptance of a Covid-19 vaccine is associated with ability to detect fake news and health literacy. J. Public Health.

[58] Tagliabue F., Galassi L., Mariani P. (2020). The “Pandemic” of Disinformation in COVID-19. SN Compr. Clin. Med..

[59] Wilson L.S., Wiysonge C. (2020). Social media and vaccine hesitancy. BMJ Glob. Health.

[60] Moscadelli A., Albora G., Biamonte M.A., Giorgetti D., Innocenzio M., Paoli S., Lorini C., Bonanni P., Bonaccorsi G. (2020). Fake news and covid-19 in Italy: Results of a quantitative observational study. Int. J. Environ. Res. Public Health.

[61] Centers for Disease Control and Prevention (CDC) (2021). Interim Clinical Considerations for Use of COVID-19 Vaccines Currently Authorized in the United States.

[62] Conti R., Akesson J., Weiss E., Sae-Hau M., Lee M., Gracia G., Connell B., Culp L., Metcalfe R. (2021). COVID-19 Vaccine Hesitancy among Blood Cancer Patients.

[63] Lasser K.E., Kim T.W., Alford D.P., Cabral H., Saitz R., Samet J.H. (2011). Is unhealthy substance use associated with failure to receive cancer screening and flu vaccination? A retrospective cross-sectional study. BMJ Open.

[64] Druss B.G., Rosenheck R.A., Desai M.M., Perlin J.B. (2002). Quality of preventive medical care for patients with mental disorders. Med. Care.

[65] Merrick E.L., Hodgkin D., Garnick D.W., Horgan C.M., Panas L., Ryan M., Saitz R., Blow F.C. (2008). Unhealthy drinking patterns and receipt of preventive medical services by older adults. J. Gen. Intern. Med..

[66] McKnight B., McKnight I., Kerr T., Li K., Montaner J., Wood E. (2006). Prevalence and correlates of cervical cancer screening among injection drug users. J. Obstet. Gynaecol. Can..

[67] Barocas J.A. (2020). Business Not as Usual—Covid-19 Vaccination in Persons with Substance Use Disorders. N. Engl. J. Med..

[68] Merrill J.O., Rhodes L.A., Deyo R.A., Marlatt G.A., Bradley K.A. (2002). Mutual mistrust in the medical care of drug users: The keys to the “narc” cabinet. J. Gen. Intern. Med..

[69] Chapman B.G., Coups E.J. (1999). Predictors of Influenza Vaccine Acceptance among Healthy Adults. Prev. Med..

[70] Bokemper S.E., Huber G.A., Gerber A.S., James E.K., Omer S.B. (2021). Timing of COVID-19 vaccine approval and endorsement by public figures. Vaccine.

[71] Jackson S.E., Paul E., Brown J., Steptoe A., Fancourt D. (2021). Negative vaccine attitudes and intentions to vaccinate against Covid-19 in relation to smoking status: A population survey of UK adults. Nicotine Tob. Res..

[72] Fisher M., Moores L., Alsharif M.N., Paganini-Hill A. (2016). Definition and Implications of the Preventable Stroke. JAMA Neurol..

[73] State of Hawaii, Department of Health, Disease Outbreak Control Division. COVID-19 Summary Metrics: Hawaii COVID-19 Daily News Digest 1 February 2021. https://health.hawaii.gov/news/covid-19/hawaii-covid-19-daily-news-digest-february-1-2021.

[74] State of Hawaii, Department of Health, Disease Outbreak Control Division COVID-19 Summary Metrics: Hawaii COVID-19. Honolulu, Hawaii. https://health.hawaii.gov/coronavirusdisease2019/current-situation-in-hawaii.

