# Supplementary material for: Variables Associated with Coronavirus Disease 2019 Vaccine Hesitancy Amongst Patients with Neurological Disorders
Source: Infect Dis Rep. 2021 Aug 30;13(3):763–810. doi: 10.3390/idr13030072 (PMC8482072; doi:10.3390/idr13030072)
Supplement: Supplementary file 1 [file idr-13-00072-s001.zip › idr-1280751-supplementary.pdf]

Supplemental Table S1. Comparison of patients providing complete or partial surveys (participants) to those who did not participate (i.e., non-response, declined, or break-offs). Relative to non-participants, Whites and married patients had a greater odds of survey participation; NAANs were at reduced odds of survey participation.

		Wilcoxon Rank Sum Test
**Age**		
Participants (n = 359)	60.00 (45.00, 71.00)	1.00 (-1.00, 3.00), p = 0.32
Declined/Non-Response (n = 1135)	58.00 (42.00, 71.00)	
**Geographic Origin Population Size**		
Participants (n = 353)	51511 (29066, 51601)	$7.94 \text{ x } 10^{-5}$ ($-2.26 \text{ x } 10^{-5}$, 90.00), p = 0.26
Declined/Non-Response (n = 1119)	51511 (31445, 51601)	
**Median Household Income**		
Participants (n = 353)	94541 (79074, 105161)	$4.08 \text{ x } 10^{-5}$ ($-1.30 \text{ x } 10^{-5}$, 14.00), p = 0.59
Declined/Non-Response (n = 1119)	93832 (77275, 105161)	
**Overall Poverty Level (Ratio) per Patient Municipality**		
Participants (n = 353)	0.056 (0.049, 0.096)	$3.91 \text{ x } 10^{-6}$ ($-3.97 \text{ x } 10^{-5}$, $3.15 \text{ x } 10^{-5}$), p = 0.91
Declined/Non-Response (n = 1119)	0.056 (0.049, 0.10)	
**Poverty Level (Ratio) for Ages 18-64 per Patient Municipality**		
Participants (n = 353)	0.059 (0.049, 0.089)	$5.54 \text{ x } 10^{-6}$ ($-3.04 \text{ x } 10^{-5}$, $9.66 \text{ x } 10^{-4}$), p = 0.73
Declined/Non-Response (n = 1119)	0.059 (0.049, 0.093)	
**Poverty Level (Ratio) for Ages 65 and Over per Patient Municipality**		
Participants (n = 353)	0.051 (0.042, 0.082)	$9.33 \text{ x } 10^{-6}$ (-$2.15 \text{ x } 10^{-6}$, $1.04 \text{ x } 10^{-3}$), p = 0.31
Declined/Non-Response (n = 1119)	0.051 (0.042, 0.082)	
		**Chi-Square Test or Fisher Exact Test**
**Sex (Odds Female to Male)**		
Participants<br>(Female, n = 195; Male, n = 164)	0.88 (0.69, 1.13)	χ^2 = 0.96, p = 0.33
Declined/Non-Response<br>(Female, n = 652; Male, n = 483)	1.14 (0.89, 1.45)	
**Patient Geographic Origin**		
Urban	0.99 (0.77, 1.27)	χ^2 = 0.00033, p = 0.99

(Participants, n = 190; Declined, n = 605)		
Suburbs (Participants, n = 156; Declined, n = 501)	0.98 (0.76, 1.25)	$\chi^2 = 0.017$, p = 0.90
Rural (Participants, n = 7, Declined, n = 13)	1.72 (0.58, 4.68)	$\chi^2 = 0.81$, p = 0.37
**Race**		
White (Participants, n = 149; Declined, n = 229)	1.46 (1.11, 1.93)	$\chi^2 = 7.67$, p = 0.0056
Asian (Participants, n = 97; Declined, n = 204)	0.90 (0.67, 1.21)	$\chi^2 = 0.44$, p = 0.51
Native Hawaiian or Other Pacific Islander (Participants, n = 78; Declined, n = 172)	0.85 (0.62, 1.16)	$\chi^2 = 0.93$, p = 0.34
Hispanic (Participants, n = 14; Declined, n = 30)	0.91 (0.44, 1.79)	$\chi^2 = 0.019$, p = 0.89
Black (Participants, n = 9; Declined, n = 23)	0.76 (0.30, 1.72)	$\chi^2 = 0.26$, p = 0.61
Native American or Alaskan Native (Participants, n = 4; Declined, n = 26)	0.29 (0.073, 0.85)	p = 0.026
**Marital Status**		
Married (Participants, n = 156; Declined, n = 378)	1.39 (1.05, 1.83)	$\chi^2 = 5.32$, p = 0.021
Single (Participants, n = 71; Declined, n = 251)	0.74 (0.54, 1.02)	$\chi^2 = 3.36$, p = 0.067
Divorced (Participants, n = 38; Declined, n = 108)	1.00 (0.66, 1.51)	$\chi^2 = 3.20 \times 10^{-29}$, p = 1.00
Widowed (Participants, n = 22; Declined, n = 81)	0.76 (0.44, 1.25)	$\chi^2 = 1.01$, p = 0.32
